# Engineering the Single Domain Antibodies Targeting Receptor Binding Motifs Within the Domain III of West Nile Virus Envelope Glycoprotein

**DOI:** 10.3389/fmicb.2022.801466

**Published:** 2022-04-01

**Authors:** Jana Hruškovicová, Katarína Bhide, Patrícia Petroušková, Zuzana Tkáčová, Evelína Mochnáčová, Ján Čurlík, Mangesh Bhide, Amod Kulkarni

**Affiliations:** ^1^Laboratory of Biomedical Microbiology and Immunology, The University of Veterinary Medicine and Pharmacy, Košice, Slovakia; ^2^Department of Breeding and Diseases of Game, Fish and Bees, Ecology and Cynology, The University of Veterinary Medicine and Pharmacy, Košice, Slovakia; ^3^Institute of Neuroimmunology, Slovak Academy of Sciences, Bratislava, Slovakia

**Keywords:** West Nile virus, single domain antibody, nanobodies, human brain microvascular endothelial cells, phage display, West Nile virus-like particles

## Abstract

West Nile virus (WNV) is a mosquito-borne neurotrophic flavivirus causing mild febrile illness to severe encephalitis and acute flaccid paralysis with long-term or permanent neurological disorders. Due to the absence of targeted therapy or vaccines, there is a growing need to develop effective anti-WNV therapy. In this study, single-domain antibodies (sdAbs) were developed against the domain III (DIII) of WNV’s envelope glycoprotein to interrupt the interaction between DIII and the human brain microvascular endothelial cells (hBMEC). The peripheral blood mononuclear cells of the llama immunized with recombinant DIII^L297–S403^ (rDIII) were used to generate a variable heavy chain only (VHH)-*Escherichia coli* library, and phage display was performed using the M13K07ΔpIII Hyperphages system. Phages displaying sdAbs against rDIII were panned with the synthetic analogs of the DIII receptor binding motifs, DIII-1^G299–K307^ and DIII-2^V371–R388^, and the VHH gene from the eluted phages was subcloned into *E. coli* SHuffle. Soluble sdAbs purified from 96 *E. coli* SHuffle clones were screened to identify 20 candidates strongly binding to the synthetic analogs of DIII-1^G299–K307^ and DIII-2^V371–R388^ on a dot blot assay. Among them, sdAb_A1_, sdAb_A6_, sdAb_A9_, and sdAb_A10_ blocked the interaction between rDIII and human brain microvascular endothelial cells (hBMECs) on Western blot and cell ELISA. However, optimum stability during the overexpression was noticed only for sdAb_A10_ and it also neutralized the WNV–like particles (WNV-VLP) in the Luciferase assay with an half maximal effective concentration (EC_50_) of 1.48 nm. Furthermore, the hemocompatibility and cytotoxicity of sdAb_A10_ were assessed by a hemolytic assay and XTT-based hBMEC proliferation assay resulting in 0.1% of hemolytic activity and 82% hBMEC viability, respectively. Therefore, the sdAb_A10_ targeting DIII-2^V371–R388^ of the WNV envelope glycoprotein is observed to be suitable for *in vivo* trials as a specific therapy for WNV–induced neuropathogenesis.

## Introduction

West Nile virus (WNV) is the most common mosquito-borne flavivirus belonging to the family of Japanese encephalitis virus serocomplex causing mild to severe disease in humans. The principal vectors of WNV are culicine mosquitos, and the main reservoir hosts are birds, participating in the worldwide distribution of the virus. Humans, horses, and other mammals are dead-end hosts (Petersen and Marfin, 2002). The genome of WNV is composed of linear positive-sense single-stranded RNA of ∼11 kb encoding a single large polyprotein. During virus replication, the polyprotein is post-translationally cleaved by both hosts and viral endoproteases into three structural proteins viz. envelope glycoprotein (E), nucleocapsid protein (C), and precursor membrane glycoprotein (prM), and seven non-structural proteins (NS1, NS2A, NS2B, NS3, NS4A, NS4B, and NS5) (May et al., 2011).

Although WNV infection in humans is subclinical, about 25% of infected cases show mild febrile illnesses, headache, generalized weakness, morbilliform or maculopapular rash, and myalgia (May et al., 2011; Petersen et al., 2013). On the other hand, a small percentage of infected cases progress into severe neuroinvasive diseases characterized by acute encephalitis, meningitis, and acute flaccid paralysis. In 2019, 443 WNV infections were reported in the EU, leading to a fatality of 52 cases (Centers for Disease Control and Prevention, 2021). Similarly, 971 cases were recorded in the United States with 9% mortality (Centers for Disease Control and Prevention, 2020). The prevalence of the neuroinvasive form is more common in elders with a mortality rate of 15–29%, and two-thirds of the survivors can experience long-term or permanent neurological disorders (Petersen and Marfin, 2002; Yao and Montgomery, 2016).

A major hurdle in the development of a specific therapy against WNV is the ability of the virus to overcome the blood–brain barrier (BBB) and propagate in the neurons and myeloid cells of the brain (Suthar et al., 2010; Winkelmann et al., 2016). The molecular mechanism underlying the BBB penetration of WNV has been studied using murine models (Wang et al., 2004; Wang P. H. et al., 2008). The most convincing explanation for WNV entry into the central nervous system (CNS) denotes the binding of E glycoprotein to the receptor of the brain microvascular endothelial cells (BMEC) and subsequent receptor-mediated transcytosis without disrupting the integrity of BBB (Verma et al., 2009; Suthar et al., 2013). Toll-like receptor 3 mediated WNV entry into the brain involving BBB permeabilization and a “Trojan horse” mechanism through infected T cells, macrophages, and polymorphonuclear neutrophils is also an important mechanism of BBB crossing (Wang et al., 2004; Wang S. H. et al., 2008; Paul et al., 2017).

On the other hand, BBB represents a significant barrier to drug delivery to the brain (Azad et al., 2015; Daneman and Prat, 2015). Intravenous infusion of human immunoglobulins containing anti-WNV antibodies had shown possibilities to ameliorate established WNV infection (Shimoni et al., 2001; Hamdan et al., 2002; Ben-Nathan et al., 2003). Thus, neutralizing monoclonal antibodies (mAbs) targeting the E glycoprotein of WNV and particularly domain III (DIII), the major viral hemagglutinin involved in receptor recognition and fusion to the host cell, have been developed (Beasley and Barrett, 2002; Vogt et al., 2009; Tsioris et al., 2015). Although antibodies are promising therapeutic tools, the major disadvantage is their size (approximately 150 kDa), which limits their penetration into the brain tissue (Steeland et al., 2016). On the other hand, single-domain antibodies (sdAbs) (also known as nanobodies) engineered from the naturally occurring heavy chain only antibodies in the serum of camelids are attractive substitutes for conventional monoclonal antibodies that can cross the BBB (Muruganandam et al., 2001; Li et al., 2012). Their unique features [as reviewed in Jovcevska and Muyldermans (2020)] include small size (12–15 kDa), high stability incurred by the presence of an interloop disulfide bond, enhanced solubility due to the presence of smaller and/or more hydrophilic amino acids (F42 or Y42, E49, R50, and G52) in CDR2, and strong antigen-binding potential acquired by fully functional and significantly shorter CDR3 (4–8 amino acids) (Jovcevska and Muyldermans, 2020). In particular, the CDR3 loop of sdAbs, possessing a finger-like structure and convex paratope, aids in reaching poorly accessible epitopes hidden in antigen cavities (Jovcevska and Muyldermans, 2020). Moreover, a high degree of similarity (∼80%) between the amino acid sequences of the human variable regions of antibodies and camelid sdAbs along with the 95% sequence identity in the immunoglobulin variable region (IgV) gene repertoire of camelids and humans has led to the low immunogenicity of sdAbs (Vu et al., 1997; Muyldermans, 2013). Therefore, sdAbs are employed in various clinical trials to treat cancers, respiratory tract infections, viral diarrhea, psoriasis, and autoimmune disease (Jovcevska and Muyldermans, 2020).

In our previous study, receptor binding motifs on the DIII of WNV E glycoprotein interacting with human brain microvascular endothelial cells (hBMCEs) were identified as DIII-1^G299–K307^ and DIII-2^V371–R388^ (Mertinkova et al., 2020). In the present study, sdAbs recognizing DIII-1^G299–K307^ and DIII-2^V371–R388^ were developed using the M13KO7ΔpIII Hyperphage system as described by us recently (Kulkarni et al., 2020). The resulting sdAbs were evaluated for toxicity, hemocompatibility, and ability to hinder the interaction between DIII and endothelial cells and neutralize entry of WNV-like particles (WNV-VLP) in the cultured cells.

## Materials and Methods

### Overexpression and Purification of Recombinant DIII (rDIII)

The DIII of the E glycoprotein of the WNV (strain goshawk, Genbank: DQ116961.1) spanning the amino acids L^297^–S^403^ was recombinantly produced. The amplification of the gene fragment, cloning into a pQE-30-mCherry-STOP vector, and transformation of *Escherichia coli* M15 are described in our earlier study (Mertinkova et al., 2020). The sequence of DIII^L297–S403^ and the vector are presented in Supplementary Figure 1. To overexpress the rDIII, a transformant was propagated in 100 ml of a TB medium [(tryptone 12 g/l; Duchefa Biochemie B.V., Haarlem, Netherlands), yeast extract (24 g/l; Duchefa Biochemie, Haarlem, Netherlands), glycerol (0.6%; MikroChem, Pezinok, Slovakia), Na_2_HPO_4_.12 H_2_O (25 mM; Sigma-Aldrich, Darmstadt, Germany), KH_2_PO_4_ (25 mM; Sigma-Aldrich)] supplemented with glucose (1%; MikroChem, Pezinok, Slovakia), kanamycin (25 μg/ml; Duchefa Biochemie, Haarlem, Netherlands), and carbenicillin (50 μg/ml; Duchefa Biochemie, Haarlem, Netherlands) at 30°C until OD_600_ = 0.6. Induction was performed with 1 mM isopropyl β-d-1-thiogalactopyranoside (IPTG) (Fermentas, Bratislava, Slovakia) in the TB medium supplemented with the above-stated antibiotics for 8 h at 30°C.

The overexpressed rDIII was purified with nickel affinity chromatography (Ni-NTA agarose beads, ABT, Madrid, Spain) followed by size exclusion chromatography (Sephadex G-25, GE Healthcare, Chicago, IL, United States) on an ÄKTA purifier (GE Healthcare) as described in our previous publication (Mertinkova et al., 2020). The purity of rDIII was verified by lithium dodecyl sulfate-polyacrylamide gel electrophoresis (LDS-PAGE), and matrix-assisted laser desorption/ionization - time-of-flight mass spectrometry (MALDI-TOF MS) (Bruker, Bremen, Germany) performed exactly as described by Mertinkova et al. (2020), and the protein was stored at −20°C until further use.

### Animal Welfare and *in vivo* Immunization

A 4-year-old healthy male llama (*Llama alpaca*) was reared at the animal farm of The University of Veterinary Medicine and Pharmacy in Košice. Immunization and blood collection were performed following the guidelines of the EU animal welfare legislation and the University’s ethical committee. The llama was immunized with rDIII of WNV along with the other antigens (rDIII of tick-borne encephalitis virus and NadA protein of *Neisseria meningitidis*) as described in our recent study (Kulkarni et al., 2020). The strategy of the immunization of the llama with multiple antigens was adopted from the protocol described by Pardon et al. (2014) with some modifications. In brief, the llama was immunized with antigens (i/m injections) for 6 weeks. The first immunization was performed with 200 μg of each protein mixed with Freund’s complete adjuvant (Sigma-Aldrich) in a ratio of 1:1 (v/v). Subsequently, five weekly immunizations were carried out with 100 μg of each protein mixed with Freund’s incomplete adjuvant (Statens Serum Institut, Copenhagen, Denmark) 1:1 (v/v). One week after the last immunization, 100 ml of blood was collected from the *vena jugularis* of the llama in 50 ml sterile falcon tubes containing 2500 IU heparin (Zentiva, Prague, Czechia).

### Amplification of Gene Fragment Encoding VHH and Generation of VHH-*E. coli* Library

Steps involving the isolation of peripheral blood mononuclear cells (PBMCs) from the blood of the immunized llama, amplification of VHH (variable domain of the heavy chain only antibodies, gene fragment encompassing framework 1-framework 4 of sdAbs), its cloning into phagemid pJB12, and subsequent transformation of *E. coli* XL-1 blue (New England Biolabs, Frankfurt, Germany) to generate the VHH-*E. coli* library were performed exactly as described in our earlier publication (Kulkarni et al., 2020). Thus, the details are described in Supplementary Method 1. Primers used to amplify VHH are presented in Table 1.

**TABLE 1:** List of primers.

No.	Description	Sequence used to design primers	Primer	Sequence 5′–3′	Amplicon length (bp)
1	Protein E domain III	DQ116961.1	Sense	ACAGGATCCCTGAAGGGAACA ACATATGGA	339
			Antisense	CTTGTCGACGCTGCTCCCAGA TTTGTGCCA	498
2	Insertom check		UA-insertom-His-sense	CGCATCACCATCACCATCACG	∼540
			UA-insertom-GFP-antisense	ACCAAAATTGGGACAACACCAGTG	
3	Reverse transcription		VHH-Not-R	CCAGCGGCCGCTSWGGAGA CRGTGACCWGGGTCC	
4	Amplification of sdAb		VHH-F	GCGGCCCAGCCGGCCGCCSAG GTGSAGGTSSWGSMGTC	∼500
			VHH-R	AAAGGCCCCCGAGGCCGATSWG GAGACRGTGACCWGGGTCC	

*Underlined nucleotides represent restriction sites. GGATCC is restriction site for BamhI, GTCGAC for SalI, and GGCCCNNNNGGCC for SfiI.*

### Phage Packaging

The VHH-*E. coli* library (initial OD_600_ of 0.1) was amplified in 800 ml of the 2xTY medium (16 g/l tryptone, 10 g/l yeast extract and 5 g/l NaCl; pH = 7.0) supplemented with tetracycline (50 μg/ml), chloramphenicol (50 μg/ml), and glucose (4%) to obtain the final OD_600_ of 0.5. The amplified VHH-*E. coli* library was superinfected with Hyperphage M13K07ΔpIII (Progen Biotechnik, Heidelberg, Germany) with a multiplicity of infection 1:20 (*E. coli*: phages). The superinfected library was incubated at 37°C for 30 min followed by centrifugation at 3,500 × *g* to remove unbound phages. The pellet was resuspended in a new 2xTY medium supplemented with tetracycline (50 μg/ml), chloramphenicol (50 μg/ml), and kanamycin (50 μg/ml) and incubated for 16 h at 37°C. The escaped phages were precipitated with PEG-NaCl (20% polyethylene glycol and 2.5 M NaCl). The precipitated phages were resuspended in a phage dilution buffer (10 mM Tris-HCl, 20 mM NaCl, 2 mM EDTA; pH = 7.3) and enumerated by spectrophotometry using the formula (*A*_269_ − *A*_320_) × 6 × 10^16^/number of bases per virion (Phage Concentration Calculator, 2012). All the chemicals used to perform phage display were procured from Sigma-Aldrich (unless stated otherwise), and the antibiotics were purchased from Duchefa Biochemie BV, Haarlem, Netherlands. These purified phages are designated as the VHH-phage library.

### Confirming the Non-infectivity of Escaped Phages

Next, 10 μl of purified phages were inoculated in a 50 ml culture of *E. coli* XL-1 blue (OD_600_ 0.45) and incubated at 37°C for 30 min. Then, 150 μl of the culture was plated on an LB (Sigma-Aldrich) agar plate supplemented with tetracycline (50 μg/ml), chloramphenicol (50 μg/ml), and kanamycin (50 μg/ml) and incubated at 37°C overnight.

### Biopanning

First, the negative adsorption was performed to eliminate the phages with non-specific affinity to plastic wear and bovine serum albumin (BSA) (AppliChem, Darmstadt, Germany) from the VHH-phage library. Briefly, 1 × 10^14^ phages were incubated in empty wells for 1 h at room temperature, the content of the well was transferred to the wells coated with 2% BSA and incubated for 1 h at room temperature. For both, negative adsorption Pierce Nickel-coated 96-well plates (Thermo Fisher Scientific, Waltham, MA, United States) were used. After incubation, the content of the wells was used for biopanning.

One microgram (78.12 pmol) of rDIII was coated on Pierce Nickel-coated 96-well plates, and the non-specific sites were blocked with 2% BSA in phosphate-buffered saline (PBS). Next, the VHH-phage library (∼1 × 10^14^ phages) was added to the rDIII-coated wells for 2 h, and 10 stringent washings with PBS containing 0.1% Tween 20 (PBST-20; Sigma-Aldrich) were performed. It is expected that specific phages remain attached to rDIII, and non-specific phages are eliminated during washings. Receptor binding motifs DIII-1^G299–K307^ and DIII-2^V371–R388^ were synthesized commercially with biotinylated C-terminal lysine (Caslo, Kongens Lyngby, Denmark) (Supplementary Table 1). These synthetic motives were used for the competitive elution of specific phages. Therefore, 10 μg (10 nM) of DIII-1^G299–K307^ reconstituted in phosphate buffer (pH = 7.0) was added. After 15 min of incubation, the flow through containing phages bound to DIII-1^G299–K307^ (competitively eluted) was collected, and the phage DNA was extracted by heat treatment (98°C, 10 min). Simultaneously, the panning was conducted to elute the phages using DIII-2^V371–R388^, and phage DNA was extracted. Note that only one round of biopanning was performed to preserve the diversity of phages.

### Production of Soluble Single-Domain Antibodies

The DNA isolated from the phages eluted in panning was used to amplify the VHH encompassing frameworks 1–4 using primers VHH-F and VHH-R (Table 1). The PCR conditions were 95°C 2 min, 25 cycles of (95°C 20 s, 56°C 30 s, 68°C 1 min), and 68°C 10 min. The amplicons were separated on agarose gel, purified with NucleoSpin (Macherey-Nagel, Düren, Germany), and digested with *Sfi*I (Thermo Fisher Scientific). The digested amplicons were ligated into the in-house modified vector pQE30-UA-mCherry-3xStop-GFP vector (Supplementary Figure 3) and electroporated into the *E. coli* SHuffle (New England Biolabs) using the manufacturer’s preset method for *E. coli* in the Gene Pulser X cell. Three electroporations were performed per elution, and the transformants were grown on two LB plates supplemented with carbenicillin (100 μg/ml) at 30°C for 16 h. Forty-eight isolated colonies for each elution (elution with DIII1^G299–K307^ or DIII-2^V371–R388^) were randomly picked and resuspended in 1 ml of TB medium supplemented with carbenicillin (100 μg/ml) in a 96 deep well plate (Merck, Darmstadt, Germany). The propagation was performed at 30°C for 16 h with constant shaking (10 × *g*) followed by centrifugation at 2630 × *g* and induction with 0.5 mM IPTG in the TB medium supplemented with carbenicillin (100 μg/ml) at 30°C for 3 h followed by 22°C for 16 h (with shaking at 10 × *g*). The induced bacterial culture was centrifuged at 2,630 × *g* for 40 min, and the resulting pellet was lysed during four freeze-thaw cycles in the presence of a lysis buffer (50 mM NaH_2_PO_4_ 2H_2_O, 300 mM NaCl, 8 M Urea, 10% glycerol, and 10 mM imidazole; pH = 8) and 10 cycles of sonication (75 Hz, 45 s pulse, and 1 min pause). The lysate was centrifuged at 23,660 × *g* for 30 min, and the supernatant was purified by nickel affinity chromatography using His-Mag sepharose Ni beads (GE Healthcare) as stated in the manufacturer’s protocol.

To confirm the presence of sdAbs in purified lysate, nine randomly selected lysates were resolved on 12% LDS PAGE and electrotransferred on nitrocellulose (NC) membrane (0.45 μm; GE Healthcare). After blocking the non-specific sites on the NC membrane with 5% BSA, Western blot was performed using HisProbe-HRP conjugate (1:5000 in TBST-20, Thermo Fisher Scientific) and SuperSignal West Dura chemiluminescent substrate (Thermo Fisher Scientific) as described in our earlier publication (Mertinkova et al., 2020).

### Interaction of Single-Domain Antibodies With Receptor Binding Motifs of rDIII (DIII-1^G299–K307^ or DIII-2^V371–R388^): Dot Blot Assay

Purified sdAbs (1 μl) were spotted on NC membranes, and non-specific binding sites were blocked with 5% BSA dissolved in tris-buffered saline (TBS; pH = 7.3, Sigma-Aldrich). After a wash with TBS containing 0.05% Tween 20 (TBST-20; 5 min), membranes were separately incubated (1 h) with either biotinylated DIII-1^G299–K307^ or DIII-2^V371–R388^ reconstituted in a phosphate buffer (1 μg/ml, pH = 7.0). After three washings, streptavidin-HRP conjugate (1:30,000 in TBST-20; Sigma-Aldrich) was added, and the membranes were incubated for 1 h. Subsequently, five washes with TBST-20 (5 min each) and one with TBS (5 min) were performed before incubating the membranes with a SuperSignal West Dura chemiluminescent substrate, and the signal was recorded on a C-DiGit Blot Scanner (Odyssey CLx, Cambridge, United Kingdom). Simultaneously, sdAbs raised against *N. meningitides* [VHH_F3_ (Kulkarni et al., 2020): non-related sdAbs; negative control] and hyperimmune serum of horse surviving natural WNV infection (positive control) spotted on NC membranes, were included in the assay to undergo incubations with DIII-1^G299–K307^ or DIII-2^V371–R388^, streptavidin-HRP conjugate, and chemiluminescent substrate, followed by signal detection.

### Human Brain Microvascular Endothelial Cells Culture and Protein Extraction

Human brain microvascular endothelial cells (hBMEC/D3 cell line; Merck/Millipore) were cultured in a T-75 cell culture flask (Sarstedt, Bratislava, Slovakia) following the protocol described by Jimenez-Munguia et al. (2018) with minor modifications. In short, 1 × 10^6^ hBMECs (second passage) were seeded on a collagen type I (Corning, Corning, NY, United States) coated T-75 cell culture flask containing 10 ml of endothelial complete medium. The complete medium contained DMEM-F12 (Thermo Fisher Scientific) supplemented with 10% fetal bovine serum, L-glutamin (2 mM; Life Technologies, Waltham, MA, United States), 1 × Pen-strep (Jena Bioscience, Jena, Germany), hydrocortisone (0.01 g/ml; Sigma-Aldrich), ascorbic acid (10 μg/ml; Sigma-Aldrich), 1 M HEPES, and bFGF (10 μg/ml; Sigma-Aldrich). The cells were grown at 37°C in a 5% CO_2_ incubator until they formed a monolayer.

Proteins from the confluent monolayer of hBMECs were extracted in native conditions as described in our earlier publications (Jimenez-Munguia et al., 2018; Mertinkova et al., 2020). The protein extract was subjected to gel filtration (in-house prepared 30 ml column, Sephadex G-25, Marshall Scientific, NH, United States) on an ÄKTA purifier (2 ml/min flow, max 0.25 MPa pressure) against PBS (pH = 7.3) as described before (Kanova et al., 2019). Protein concentration was measured by Bradford assay, and the purified proteins were aliquoted and stored at −80°C until further use.

### Blocking of Interaction Between rDIII and Human Brain Microvascular Endothelial Cell Proteins by Single-Domain Antibodies

The specific interaction of rDIII with the ∼15 kDa receptor of the hBMECs has been previously demonstrated by Western blotting (Mertinkova et al., 2020). In the present study, rDIII was preincubated with purified sdAbs and allowed to interact with the receptor of hBMECs. In brief, 400 μg of hBMEC proteins were fractionated on LDS-PAGE (10 cm one well gel) and electrotransferred on an NC membrane. The membrane was then sliced to generate 2 mm vertical strips. In a pilot study, various concentrations of rDIII (0.65–5 μg resuspended in TBS) were incubated with NC membrane strips having proteins of hBMECs for 3 h, and after three washings with TBST-20, the strips were incubated with HisProbe-HRP conjugate (1:5000 in TBST-20) for 30 min at room temperature. After five washings with TBST-20 (5 min each) and one with TBS (5 min), the interaction was detected with SuperSignal West Dura chemiluminescent substrate to pick up the visible interaction of rDIII and ∼15 kDa receptor.

Thereafter, 5 μg (390 pmol) of rDIII was preincubated with 5.5 μg (∼390 pmol) of sdAbs recognizing either DIII-1^G299–K307^ or DIII-2^V371–R388^ in TBS (pH = 7.3) for 90 min at room temperature with constant shaking. Simultaneously, strips with hBMEC proteins were blocked with 5% BSA in TBS for 1 h, and then preincubated rDIII was added for 3 h at room temperature with constant shaking. After three washes with TBST-20 (5 min each), strips were incubated with HisProbe-HRP conjugate (1:5000 in TBST-20) for 30 min at room temperature. Last, five washes with TBST-20 (5 min each) and one wash with TBS (5 min) were performed before incubating the strips with a SuperSignal West Dura chemiluminescent substrate. Signals were recorded on C-DiGit Blot Scanner (Odyssey CLx). As a negative control, strips were incubated with the mix of sdAbs (without rDIII), whereas strips incubated with rDIII (without preincubation) served as a positive control.

### On Cell ELISA to Confirm the Blocking Ability of Single-Domain Antibodies

The hBMEC were seeded (fourth passage, 1 × 10^4^ cells/well) on a collagen type I–coated 96-well plate (tissue culture flat bottom plate, TPP, Trasadingen, Switzerland) containing 200 μl of endothelial complete medium. After reaching 80% confluency, the cells were washed with Dulbecco PBS (pH = 7.3, Thermo Fisher Scientific) and fixed with 4% paraformaldehyde (Sigma-Aldrich) for 15 min at room temperature. The fixed cells were again washed with Dulbecco PBS and blocked with 5% BSA in PBS for 1 h at room temperature. Simultaneously, 5 μg (390 pmol) of rDIII was preincubated with 5.5 μg (∼390 pmol) of sdAbs (sdAb_A1_, sdAb_A6_, sdAb_A9_, and sdAb_A10_) for 1.5 h at room temperature with constant shaking. Thereafter, the rDIII preincubated with sdAbs was added on fixed hBMEC in 200 μl of PBST-20 and 1% BSA for 3 h at room temperature with constant shaking. After three washings with PBST-20 (5 min each), the cells were incubated with HisProbe-HRP conjugate (1:5000 in PBST-20) for 1 h, and stringent washings with PBST-20 (5 times, 5 min each) and PBS (5 min) were performed. Then, 100 μl of 1-Step Ultra TMB-ELISA substrate (Thermo Fisher Scientific) was added, incubated for 20 min, and then the contents of wells were transferred to a new microtiter ELISA plate (Thermo Fisher Scientific). The reaction was stopped with 2 M H_2_SO_4_, and the absorbance was measured at 450 nm on an ELISA plate reader (GMI-Trusted Laboratory Solutions, Ramsey, NM, United States). For the positive and negative controls, fixed hBMECs were incubated with either the rDIII (without sdAb) or sdAb (without rDIII), respectively. Note that the entire assay was performed in six replicates, and the absorbance values in each treatment were compared for statistical significance (*p* < 0.05) on Prism v.5 (GraphPad Software, Inc., San Diego, CA, United States) using one-way ANOVA and Bonferroni’s *post hoc* test.

### Large-Scale Production of Single-Domain Antibodies

Transformants from the glycerol stock carrying sdAb_A1_, sdAb_A6_, sdAb_A9_, and sdAb_A10_ were propagated in 200 ml of TB medium, and proteins were overexpressed with IPTG as described. The bacterial lysate was subjected to nickel affinity chromatography using Ni-NTA agarose beads, and purified sdAbs were dialyzed against 1× PBS (pH = 7.3) using Spectra/Por Dialysis Membrane (Molecular weight cutoff: 3.5–5 kDa, Spectrum Labs, Phoenix, AZ, United States) at 4°C (overnight dialysis). The quality of sdAbs was assessed by LDS-PAGE, and the molecular weight was assessed by MALDI-TOF MS (Bruker) as described.

Simultaneously, the VHH region within the transformants producing sdAb_A1_, sdAb_A6_, sdAb_A9_, and sdAb_A10_ was sequenced using UA-insertom-His-sense and UA-insertom-GFP-antisense primers (Table 1).

### Neutralization of Virus-Like Particle

Human embryonic kidney 293 cells (HEK293/17, passage 11) and custom synthesized virus-like particles (VLP) possessing C, M, and E proteins of WNV along with the luciferase reporter *Fluc* gene (WNV-VLP) were gifted by Axon Neuroscience, Bratislava, Slovakia. First, the titer of WNV-VLP was determined following the recently published protocol (Nie et al., 2020). Note that the titration of WNV-VLP was shared for the present study and our recently published study (Mertinková et al., 2021); therefore, the details are described in Supplementary Method 2. For the neutralization assay, HEK293/17 cells were cultivated in a 96-well plate for 16 h. Thereafter, the ability of sdAb_A1_, sdAb_A6_, and sdAb_A10_ to block the entry of WNV-VLP into the HEK293/17 cells was assessed by a luciferase assay system (Promega Madison, WI, United States) as described in the published protocol (Nie et al., 2020). Additionally, the hyperimmune serum of a horse surviving natural WNV infection was included in the assay as a positive control, whereas the VHH_*F*3_ [non-related sdAb: raised against *N. meningitidis* (Kulkarni et al., 2020)] and sdAb_A3_ (presumed to be non-neutralizing sdAb) were included as negative controls. Details of the experimental procedure are described in Supplementary Method 3.

### Assessing the Cross-Reactivity of Single-Domain Antibodies to rDIII of Tick-Borne Encephalitis Virus

The cross-reactivity of sdAb_A1_, sdAb_A6_, and sdAb_A10_ to bind rDIII^G301–K395^ of tick-borne encephalitis virus (TBEV) (accession no. 2022145A) was assessed on ELISA. Next, 5 μg (406.8 pmol) of TBEV-rDIII^G301–K395^ resuspended in coating buffer (0.5 M Na_2_CO_3_, 0.5 M NaHCO_3_, pH 9.5) was coated on the wells of the ELISA plate (overnight at 4°C), and the non-specific binding sites were blocked with 5% BSA in PBS (1 h). Next, 5.5 μg (∼390 pmol) of either sdAb_A1_, sdAb_A6_, or sdAb_A10_ resuspended in PBST-20 and 1% BSA were added (1 h). After three washings with PBST-20 (5 min each), anti-Myc antibody conjugated with HRP (1:10,000 in PBST-20; Abcam, Cambridge, United Kingdom) was added (1 h), and the chromogenic reaction was developed with a 1-Step Ultra TMB-ELISA substrate. After 25 min, the reaction was stopped with 2 M H_2_SO_4_, and the absorbance was measured at 450 nm on the ELISA plate reader. For the positive control, microtiter wells coated with 5 μg (390 pmol) of WNV- rDIII^L297–S403^ were blocked with 5% BSA in PBS (1 h), and the aforementioned amount of sdAb_A10_ (1 h) was added. Interaction between the rDIIIs of WNV/TBEV and HisProbe-HRP conjugate (1:5000 in PBST-20) formed the input controls. Whereas for negative controls, either the sdAbs or recombinant ligands (rDIIIs of WNV/TBEV) were excluded. Note that the entire assay was performed in duplicate.

### Hemolytic Assay

sdAb_A10_ was evaluated for its hemocompatibility using the method described in Rahman and Ochiai (2018) with minor modifications. In brief, 30 ml of healthy sheep blood was collected in the presence of 2,500 IU heparin (Zentiva) by a licensed veterinarian at the animal rearing house of The University of Veterinary Medicine and Pharmacy, Košice. The blood was centrifuged at 657 × *g* for 5 min, and the separated erythrocytes were washed three times with 0.9% saline and redispersed in 150 ml of 0.9% saline. Erythrocyte suspension (1 ml) was incubated with 5 μg (357 pmol), 10 μg (714 pmol), or 30 μg (2.14 nmol) of sdAb_A10_ for 1, 3, and 5 h at 37°C. Likewise, 1 ml of erythrocyte suspension either mixed with 2% Triton X-100 (Sigma) or 0.9% saline serving as positive and negative controls, respectively, were also incubated for the aforestated time points. All the samples were gently shaken at an interval of 30 min during incubations. At the end of the incubation period, the erythrocytes were separated by centrifugation (657 × *g* for 5 min), and the supernatant was kept at room temperature to oxidize hemoglobin. Last, the absorbance of oxyhemoglobin in all the samples (1 ml) was measured at 414 nm on NanoDrop One*^C^* UV-Vis Spectrophotometer (Thermo Fisher Scientific). Hemolysis in erythrocytes was calculated using the formula:% hemolysis = (absorbance_sample_ − absorbance_negative control_)/(absorbance_positive control_ − absorbance_negative control_) × 100. The entire assay was performed in triplicate.

### Toxicity Assay of sdAb_A10_

Cytotoxicity of sdAb_A10_ was determined through a cell proliferation assay kit (XTT, AppliChem) following the manufacturer’s instructions as described in our earlier publication (Mertinková et al., 2021). Briefly, hBMECs were cultivated in a 96-well plate (TPP) as described earlier. At 70% confluence, the cells were added with 2 μg (143 pmol) of sdAb_A10_ diluted into 100 μl of endothelial medium containing DMEM-F12 supplemented with 10% fetal bovine serum, L-glutamin (2 mM), 1 × Pen-strep (Jena Bioscience), 1 M HEPES, and bFGF (10 μg/ml; Sigma-Aldrich). The hBMECs incubated with 0.01% Triton X-100 served as a positive control, whereas the untreated cells served as a negative control, and wells without cells but with endothelial complete medium served as blank. After 24 h of incubation (37°C, 5% CO_2_), each well was added with 50 μl of the XTT reagent, and the incubation was extended for an additional 3 h. Thereafter, the absorbance was measured at 450 nm on an ELISA plate reader, and the viability of cells treated with sdAb_A10_ was determined by the formula (absorbance_sample_ − average of absorbance_blank_)/(average of absorbance_negative control_ − average of absorbance_blank_) × 100. The entire assay was conducted in six replicates.

## Results

### Overexpressed rDIII Used for Immunization

**FIGURE 1:**
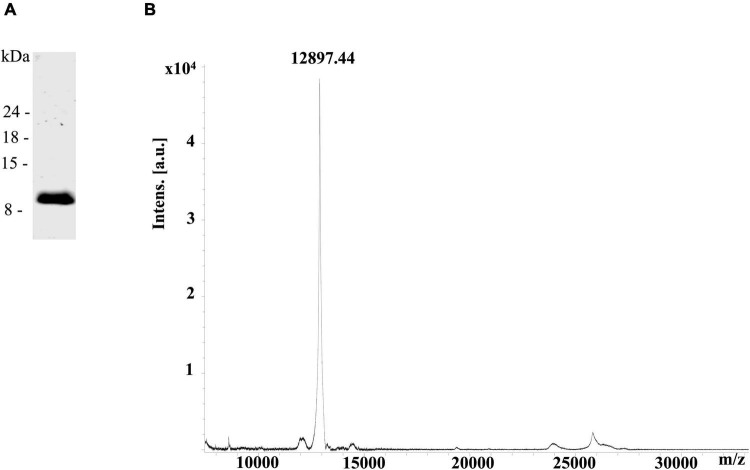
Overexpression of recombinant domain III (rDIII). **(A)** LDS-PAGE showing a single protein band of purified rDIII at ∼12 kDa. **(B)** The protein mass spectrum of rDIII showed a single peak at 12.8 kDa on MALDI-TOF MS.

The rDIII of the WNV envelope protein spanning the amino acids L^297^–S^403^ was purified by nickel affinity chromatography and size exclusion chromatography. The purity of rDIII was verified by the presence of a single protein band at ∼12 kDa on the LDS-PAGE (Figure 1A) and a protein mass spectrum of 12.8 kDa on the MALDI TOF MS (Figure 1B). The predicted molecular mass of rDIII (Genious pro software) matched with the observed protein mass on MALDI TOF MS. In total, 700 μg of purified rDIII was used to immunize the llama in six weekly injections as described in the methods section.

### VHH-*E. coli* Library and Phage Display

The llama was immunized with multiple antigens of neuroinvasive pathogens, including rDIII of WNV. Therefore, the VHH-*E. coli* library used to produce sdAbs targeting DIII of WNV in the present study and our earlier published study (Kulkarni et al., 2020) was identical. The amino acid sequence of 15 randomly selected transformants from the VHH-*E. coli* library (NCBI accession number MW286772 to MW286786) was aligned to create a distance matrix plot denoting unique sequences (Supplementary Figure 2). Most VHH sequences were unique except the clone set 2, 6, 7, and clones 10, 11. The VHH-*E. coli* library superinfected with Hyperphage M13K07ΔpIII produced 2 × 10^14^ phages per ml.

The *E. coli* XL-1 blue challenged with escaped phages (2 × 10^12^ phages) and plated on an LB agar plate supplemented with tetracycline, chloramphenicol, and kanamycin showed no growth of bacteria (zero colonies). Therefore, the phages from the VHH-phage library were asserted as non-infectious.

**FIGURE 2:**
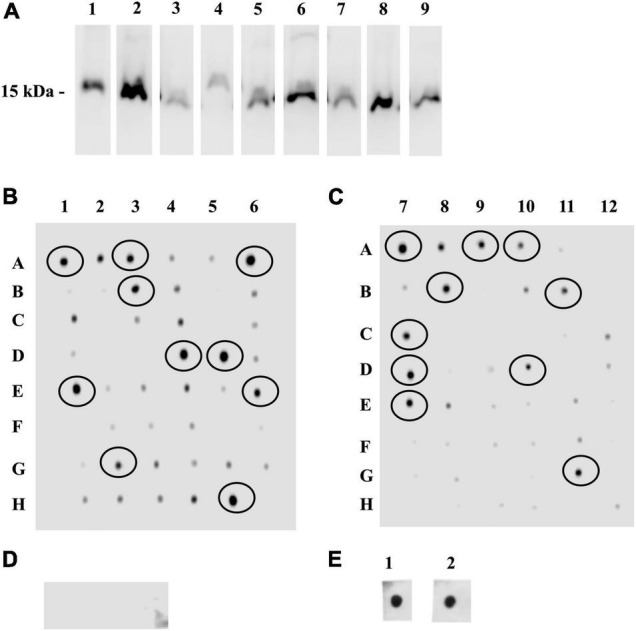
Single-domanin antibodies (sdAbs) targeting rDIII and its interaction with the synthetic analogs of DIII-1^G299–K307^ and DIII-2^V371–R388^. **(A)** Western blot assay showing chemiluminescent horseradish peroxidase (HRP) signal on the lysates of sdAb producing *Escherichia coli* SHuffle clones at ∼15 kDa incubated with HisProbe HRP conjugate. **(B–D)** Dot blot assay identifying the interaction between Ni affinity-purified 96 sdAbs targeting either the synthetic analogs of DIII-1^G299–K307^ **(B)** or DIII-2^V371–R388^ **(C)** by the streptavidin-HRP conjugate. sdAbs possessing strong interaction with synthetic analogs are circled. **(D)** non-related sdAb (raised against NadA of *N. meningitides*) used as a negative control showed no interaction with synthetic analogs of DIII-1^G299–K307^ or DIII-2^V371–R388^ in the same dot blot assay—no signals for streptavidin-HRP conjugate. **(E)** Hyperimmune serum of horse-surviving natural WNV infection incubated with DIII-1^G299–K307^ or DIII-2^V371–R388^ was used as a positive control—prominent signals for streptavidin-HRP conjugate.

The VHH-phage library was panned against synthetic analogs of DIII-1^G299–K307^ and DIII-2^V371–R388^, and the bound phages were competitively eluted. The VHH region (∼500 bp) amplified from the eluted phages (Supplementary Figure 3A) was cloned into an expression vector (Supplementary Figure 3B), and the ligated product was electroporated into the *E. coli* SHuffle. In total, 96 clones were randomly picked (48 clones against DIII-1^G299–K307^ and 48 clones against DIII-2^V371–R388^), and expression of sdAbs was induced. The presence of sdAb was confirmed by Western blot analysis in nine randomly selected clones (Figure 2A).

### Single-Domain Antibodies Interacting With Receptor Binding Motifs of rDIII: Dot Blot Assay

A dot blot assay was performed to screen the binding affinity of sdAbs to DIII-1^G299–K307^ and DIII-2^V371–R388^. A majority of the purified sdAbs interacted with DIII-1^G299–K307^ and DIII-2^V371–R388^. Twenty clones recognizing DIII-1^G299–K307^ or DIII-2^V371–R388^ were selected for further assays on the basis of the strong interaction (Figures 2B,C). The specificity of the assay was confirmed by the absence of interaction between non-related sdAbs (VHH_F3_ raised against *N. meningitidis*) and DIII-1^G299–K307^ and DIII-2^V371–R388^ (Figure 2D). Whereas the hyperimmune serum of the horse (positive control) showed a strong interaction with DIII-1^G299–K307^ and DIII-2^V371–R388^ in the same dot blot assay (Figure 2E).

### Single-Domain Antibodies Blocks the Interaction Between rDIII and Human Brain Microvascular Endothelial Cell Proteins on Western Blot

**FIGURE 3:**
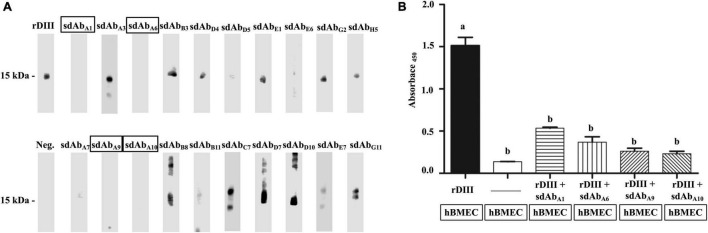
sdAb targeting the DIII blocked the interaction between rDIII and human brain microvascular endothelial cells (hBMECs) proteins. **(A)** NC strips transblotted with hBMEC proteins were incubated with either rDIII (positive control) or rDIII pretreated with sdAbs targeting receptor binding motifs of DIII. sdAb used to pretreat rDIII are mentioned on each NC strip. The interaction between rDIII/pre-treated rDIII and hBMEC proteins at ∼15 kDa was detected by HisProbe-HRP conjugate and chemiluminescent HRP substrate in Western blot analysis. sdAbs blocking the rDIII-hBMEC interaction showed the absence of chemiluminescent HRP signal at 15 kDa is framed. For negative control (Neg.), NC strip of hBMEC protein was precluded with the incubation of rDIII in the same Western blot assay, and no signal was detected. **(B)** The ability of sdAb_A1_, sdAb_A6_, sdAb_A9_, and sdAb_A10_ to block the interaction between rDIII and hBMECs was detected through cell ELISA. rDIII alone (positive control) or aforestated sdAbs-treated rDIII was incubated on paraformaldehyde-fixed hBMECs on the ELISA plate. The interaction was detected by HisProbe-HRP conjugate and UltraTMB-ELISA substrate. hBMECs precluded with the addition of either rDIII or sdAbs served as a negative control. Antigens and sdAbs added on fixed hBMECs in ELISA plate (framed) are mentioned below each bar graph. A significant difference between the compared treatments by one-way ANOVA (*p* < 0.05) is indicated by different alphabets.

Twenty sdAbs were further screened for their ability to mask rDIII and inhibit the interaction between the rDIII and ∼15 kDa hBMEC protein. At least 5 μg (0.4 nmol) of rDIII was necessary to visualize the interaction between the rDIII and hBMEC’s receptors on the Western blot (results of the pilot study). Therefore, 5 μg of rDIII was separately preincubated with an equimolar quantity of 20 shortlisted sdAbs and then allowed to interact with hBMEC proteins immobilized on the NC membrane in Western blotting. Two sdAbs, sdAb_A1_, sdAb_A6_, targeting DIII-1^G299–K307^ and two sdAbs, sdAb_A9_, sdAb_A10_, targeting DIII-2^V371–R388^ were able to block the interaction between rDIII and hBMEC’s receptors (Figure 3A).

### On Cell ELISA to Confirm the Blocking Ability of Single-Domain Antibodies

The competence of the selected sdAbs (sdAb_A1_, sdAb_A6_, sdAb_A9_, and sdAb_A10_) to block the interaction of rDIII with hBMECs was assessed by a cell ELISA. The rDIII preincubated with sdAbs were separately added on the formaldehyde-fixed hBMECs. The interaction with the fixed hBMECs was detected with a HisProbe-HRP conjugate and TMB substrate. All four sdAbs targeting either DIII-1^G299–K307^ or DIII-2^V371–R388^ significantly diminished the rDIII-hBMEC interaction (Figure 3B) whereas the unblocked rDIII (positive control) interacted optimally with fixed hBMECs. The absence of rDIII or sdAbs in the same assay produced an imperceptible signal.

### Large-Scale Production of Single-Domain Antibodies

**FIGURE 4:**
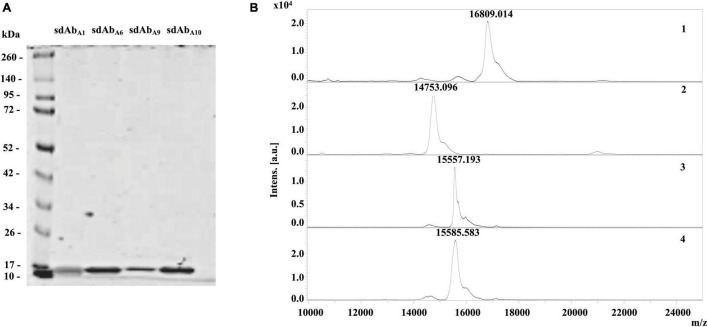
Quality assessment and amino acid sequence of overexpressed sdAbs. **(A)** Overexpressed and Nickel Nitrilotriacetic Acid (Ni NTA) purified sdAbs, sdAb_A1_, sdAb_A6_, sdAb_A9_, and sdAb_A10_, subjected to LDS-PAGE showed prominent protein band at ∼15 kDa. **(B)** Protein mass spectrum of sdAb_A1_ (1), sdAb_A6_ (2) sdAb_A9_ (3), and sdAb_A10_ (4) showing single peak on MALDI TOF MS are presented with the corresponding molecular mass of sdAbs.

Overexpressed and nickel affinity-purified sdAb_A1_, sdAb_A6_, sdAb_A9_, and sdAb_A10_ showed the presence of a single protein band in the LDS PAGE (Figure 4A). The observed molecular mass of sdAb_A1_ (16.80 kDa), sdAb_A6_ (14.76 kDa), sdAb_A9_ (15.56 kDa), and sdAb_A10_ (15.59 kDa) on MALDI-TOF MS (Figure 4B) matched with the expected molecular mass determined by Genious pro software. In large-scale production and purification, sdAb_A1_ and sdAb_A6_ showed an extreme amount of precipitation during buffer exchange. Therefore, sdAb_A1_ and sdAb_A6_ were not examined for their hemocompatibility and possible cytotoxicity assays.

During the alignment of amino acid sequences of sdAb_A1_, sdAb_A6_, sdAb_A9_, and sdAb_A10_, it was realized that sdAb_A9_ and sdAb_A10_ are identical sequences (Supplementary Figure 4). Therefore, further assays were carried out using just the sdAb_A10_.

### TCID_50_ and Virus Neutralization Test

**FIGURE 5:**
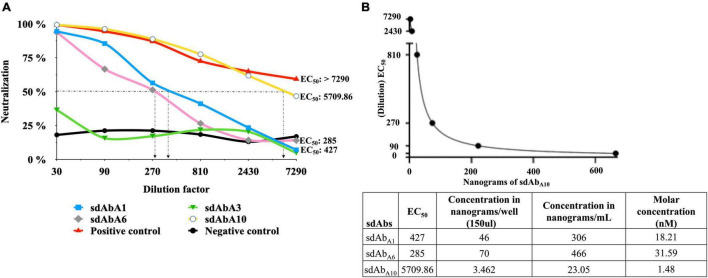
Neutralization of West Nile virus (WNV)-like particles (WNV-VLP) by sdAbs. **(A)** virus neutralization test used to assess the ability of sdAb_A1_, sdAb_A6_, and sdAb_A10_ to neutralize the WNV-VLP. Hyperimmune serum of horse (positive control), sdAb_A3_ (sdAb that does not block the binding of DIII to hBMEC), and non-related sdAb (negative control – VHH_F3_ raised against *N. meningitidis*) were used in the same assay. **(B)** The amount of sdAb (nanograms) required to neutralize the WNV-VLP (EC_50_) was calculated using non-linear regression (dilution of sdAb on *Y*-axis and quantity sdAbs in nanograms on *X*-axis) in Graphpad Prism 8 software. The concentration of sdAb in nanograms per well, per ml, and corresponding molar concentrations are tabulated.

Titration of WNV-VLP using the Luciferase assay system revealed the TCID_50_/mL to be 8,100 (Supplementary Data Sheet 2). The ability of sdAbs and the hyperimmune serum of horses to block WNV (pseudo) infection was assessed by a virus neutralization test (VNT) using VLP concentration 400–500 TCID_50_/mL. sdAb_A10_ showed the highest ability to neutralize the WNV-VLP with an EC_50_ of 5709.86 corresponding to 1.48 nM (Figure 5 and Supplementary Data Sheet 3). However, diluting the sdAb_A10_ beyond 7,290 results in a drastic reduction of WNV-VLP neutralization (Supplementary Figure 7). The other two sdAbs, sdAb_A1_, and sdAb_A6_, could neutralize the WNV-VLP with an EC_50_ of 427 and 285, which corresponds to 18.21 and 31.59 nM, respectively (Figure 5 and Supplementary Data Sheet 4), whereas the sdAb_A3_ and VHH_*F*3_ (negative control) did not neutralize the WNV-VLP (Figure 5A and Supplementary Data Sheet 4). The hyperimmune serum of the horse (positive control) showed an EC_50_ of more than 7290 (Figure 5A and Supplementary Data Sheet 3).

### Cross-Reactivity of Single-Domain Antibodies With rDIII of Tick-Borne Encephalitis Virus

**FIGURE 6:**
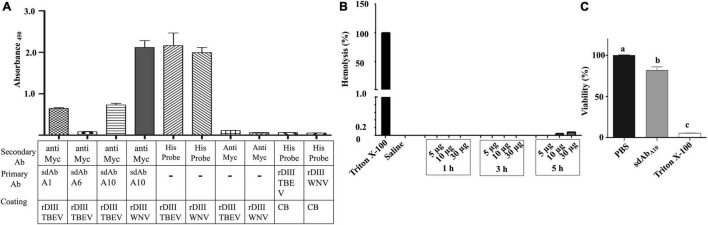
Cross-reactivity, hemocompatibility, and safety of sdAb_s_. **(A)** the interaction between sdAbs and rDIII of TBEV was assessed on ELISA to determine possible cross-reactivity. Antigen-coated (Coating), primary (primary Ab), and secondary (secondary Ab) antibodies used in the ELISA are mentioned under each bar graph of Absorbance_450_ values (Mean + SE). CB, coating buffer; Anti Myc, antiMyc antibody; HisProbe, HisProbe HRP conjugate. **(B)** Percentage of hemolysis in sheep erythrocytes caused by 5–30 μg of sdAb_A10_ was determined by measuring the absorbance of oxyhemoglobin at 414 nm over a period of 1–5 h. Even at the highest concentration of sdAb_A10_, just 0.1% hemolysis was observed as compared to 0 and 100% of hemolysis in 0.9% saline (negative control) and 2% Triton X (positive control) treated erythrocytes, respectively. The entire assay was performed in triplicate, and the bar graphs represent mean ± SE. **(C)** XTT based proliferation assay on hBMECs treated with either 2 μg (143 pM) of sdAb_A10_, 1 × PBS (negative control), and 0.1% Triton X-100 (positive control) was performed in triplicate. hBMECs treated with sdAb_A10_ showed 82% viability as compared with 100% viability in negative control and 5% viability in positive control groups. The data represent mean + SE absorbance values measured at 450 nm on an ELISA plate reader. The significant difference between the compared treatments by one-way ANOVA (*p* < 0.05) is indicated by different alphabets.

The interaction of sdAb_A1_, sdAb_A6_, and sdAb_A10_ with rDIII of TBEV, in terms of cross-reactivity was assessed on ELISA. The least cross-reactivity was observed for sdAb_A6_ (*A*_450_ = 0.08) followed by sdAb_A1_ (*A*_450_ = 0.64) and sdAb_A10_ (*A*_450_ = 0.72) (Figure 6A). Input controls showing conspicuous interaction with HisProbe-HRP conjugate (*A*_450_ = 2.0–2.16) and negative controls producing negligible signal (*A*_450_ = 0.05–0.1) affirmed the specificity of the assay.

### Hemocompatibility and Non-toxicity of sdAb_A10_

The hemocompatibility and non-toxicity of sdAb_A10_ on host cells were tested using sheep erythrocytes and hBMECs. Up to 30 μg (2.14 nmol) of sdAb_A10_ incubated with sheep erythrocytes for 5 h resulted in 0.1% hemolysis. On the other hand, the addition of 2% Triton X-100 or 0.9% saline to sheep erythrocytes produced 100% and 0% hemolysis, respectively (Figure 6B).

The cytotoxic potential of sdAb_A10_ tested by the XTT proliferation assay (Figure 6C) shows that hBMEC treated with 2 μg (143 pmol) of sdAb_A10_ showed 80% viability as compared with the 5% viability in cells treated with 0.01% Triton X-100 (positive control). As expected, 100% viability of hBMECs was noticed in PBS (negative control) treated cells.

## Discussion

With the absence of a specific antiviral medication or vaccine, supportive therapy remains the only option to treat WNV infection in humans (Petersen et al., 2013; Saiz, 2020). Although the mechanisms underlying the neuroinvasion of WNV are not fully known, virus entry into the host cells has been implicated by the attachment of E glycoprotein to host receptor molecules such as DC-SIGNR and several glycosaminoglycans (Lee et al., 2004; Wang et al., 2004; Davis et al., 2006). Subsequent internalization of WNV occurs through clathrin-mediated endocytosis, leading to the acidification of the endocytotic vesicles, the fusion of the viral envelope to the endocytic vesicle membrane, and the release of the nucleocapsid into the cytoplasm (Chu and Ng, 2004a). The ssRNA and dsRNA of WNV generated during the replication are recognized by the Toll-like receptor 7 (TLR7) and TLR3 of the host cells eliciting a systemic concentration of TNF-α, consequently leading to increased permeability of the BBB and enhanced entry of the virus into the CNS (Town et al., 2009). E glycoprotein consists of three structurally distinct domains, DI, DII, and DIII, which are connected to the viral membrane by a helical anchor (Wengler and Wengler, 1989; Zhang et al., 2017). The DI forms a bridge between DII and DIII, a peptide linker between DI and DII forms a hinge that promotes host cellular membrane fusion, and the DIII starches out of the smooth viral surface to play a crucial role in binding with cell surface receptors (Zhang et al., 2017). In particular, four residues, S^306^, K^307^, T^330^, and T^332^, located in the exposed surface on the lateral tip of DIII are critical for receptor binding (Beasley and Barrett, 2002; Oliphant et al., 2005) as the monoclonal antibodies targeting these four residues have elicited strong neutralization of WNV (Beasley and Barrett, 2002; Nybakken et al., 2005). In our previous study, two discontinuous regions of rDIII, G^299^-K^307^ and V^371^-R^388^, were identified as the motifs binding to potential receptors of hBMCEs (Mertinkova et al., 2020). Thus, in the present study, sdAbs are engineered to specifically target the identified motifs. Alternatively, DI, DII, or the fusion loop located at the tip of DII could have been targeted to produce sdAbs as neutralizing antibodies or antibody fragments (scFvs) are reported recognizing the aforestated domains of E glycoprotein (Crill and Chang, 2004; Gould et al., 2005; Oliphant et al., 2006). However, the epitopes of the fusion loop are highly conserved among flaviviruses, and 85% of mAbs targeting the fusion loop show cross-reactivity to other flaviviruses, e.g., Dengue virus (Crill and Chang, 2004; Barzon et al., 2015). Furthermore, mAbs raised against DI and DII show a low potential to neutralize WNV even at very high concentrations (Oliphant et al., 2006). Because DIII is structurally less conserved among flaviviruses (Schneeweiss et al., 2011; Ulbert, 2019), highly specific neutralizing antibodies can be raised. Such DIII targeting antibodies against WNV (CR4374) (Throsby et al., 2006) Dengue virus (Ab513) (Robinson et al., 2015), and Zika virus (ZKA64) (Stettler et al., 2016) have entered preclinical trials. In this line, in this study, we used rDIII^L297^ ^–S403^ in biopanning to engineer sdAbs.

The DIII^L297–S403^ encompasses all important amino acids (mainly S^306^, K^307^, T^330^, and T^332^) localized on the lateral tip of protein E and is involved in the cell surface receptor interaction (Supplementary Figure 1). The same was used in this study to immunize the llama to obtain specific neutralizing sdAbs. It is noteworthy that immunization of the llama was performed with two more antigens (Neisseria adhesin A, NadA, and TBEV-DIII^ G301–K395^). The protocol of immunization established by Pardon et al. (2014), adopted in the present study, encourages immunization with multiple antigens at the same time rather than consecutive immunizations.

The VHH-*E. coli* and VHH-phage library generated by this strategy contain antibodies against all used antigens. The advantages of this strategy are lesser time, cost, and labor and minimized use of animals in experiments. Specific nanobodies from such a library can be obtained either by using small targets immobilized on solid phase in biopanning (e.g., peptide, RBD only) or by using small peptides for competitive elution (e.g., motifs used in our study).

Phage display is one of the most widely used methods to produce engineered therapeutic antibodies (monoclonal, single chain, sdAbs, etc.) for cancer, degenerative disease, and several infectious diseases (Alfaleh et al., 2020). The sdAbs are better performers than conventional monoclonal antibodies due to their small size, strong antigen affinity, ability to access hard-to-reach or poorly vascularized tissues, lower toxicity, and simple production method (Comor et al., 2016; Jovcevska and Muyldermans, 2020). Hitherto, several combinations of phagemids and helperphage systems have been used for phage packaging and display of the sdAbs on either pIII or pVIII of M13 bacteriophage (Yan et al., 2014; Peltomaa et al., 2019; Salhi et al., 2020; Muyldermans, 2021). Hyperphages-M13K07ΔpIII used for packaging in the present study do not possess gene encoding pIII within their genome (Rondot et al., 2001), whereas the VHH-*E. coli* library possesses a phagemid pJB12 encoding a VHH gene fused to only the C terminal domain of pIII (supershort version of pIII, Supplementary Figure 8). Thus, the packaged phages should be displaying the sdAbs-fused CT domain without the N1 and N2 domains of PIII. The presence of the CT domain ensures proper phage assembly and their release from *E. coli* (Carmen and Jermutus, 2002). However, the absence of N1 and N2 domains inhibits the binding ability of phages to membrane protein ToIA and F pilus of *E. coli*, respectively (Lubkowski et al., 1999; Carmen and Jermutus, 2002; Paschke, 2006). Therefore, the packaged phages compulsorily display sdAbs but remain non-infectious (Paschke, 2006). The same was confirmed in the present study with the non-infectivity assay wherein *E. coli* XL1 blue incubated with escaped phages did not propagate on LB plates supplemented with tetracycline, chloramphenicol, and kanamycin.

In the present study, biopaning was performed by incubating the VHH-phage library on an rDIII-coated Ni plate, and the bound phages (withstanding rigorous washings) were eluted by 10 molar access of DIII-1^L299–K307^ and DIII-2^V371–R388^ synthetic analogs (Supplementary Table 1). This technique of competitive elution can efficiently separate phages with reasonable specificity for well-defined epitopes (Meulemans et al., 1994; Lunder et al., 2008; Thom et al., 2018). We deliberately adopted the competitive elution to obtain the sdAb phages that specifically recognize receptor binding motifs DIII-1^L299–K307^ and DIII-2^V371–R388^. Moreover, a single round of biopanning was performed to maintain the higher yield of packaged Hyperphages and to preserve the diversity of the phage, which tends to decrease with the increasing rounds of biopanning (Derda et al., 2011; ’t Hoen et al., 2012; Seidel-Greven et al., 2021). Further, a single round of panning is shown to produce sdAbs (specific binders) against NadA of *N. meninigitids* in our previous study (Kulkarni et al., 2020). As expected, more than 60% of sdAb candidates (*n* = 96) produced in this study specifically recognize DIII-1^L299–K307^ or DIII-2^V371–R388^ peptides (Figures 2B,C). Among those 96 sdAb clones, 20 sdAb showed strong interaction with receptor binding motifs (Figures 2B,C). Therefore, those sdAbs were further assessed for their ability to block the interaction between rDIII and hBMEC. Four sdAbs (sdAb_A1_, sdAb_A6_, sdAb_A9_, and sdAb_A10_) were able to block the interaction between rDIII and ∼15 kDa receptor of hBMEC (Figure 3A). The binding of the protein E to the receptor on the endothelial cells was experimentally shown recently (Mertinkova et al., 2020). A significant reduction in the binding of rDIII on cultured hBMECs was also seen in quantitative cell ELISA (Figure 3B). It is noteworthy that masking either of the receptor-binding motifs (DIII-1^L299–K307^ or DIII-2^V371–R388^) results in the complete inhibition of the interaction between rDIII and ∼15 kDa receptor of hBMEC. Studies performing mutation inhibition (Beasley and Barrett, 2002; Volk et al., 2004) and molecular docking have clarified that epitopes recognized by the most appreciated neutralizing mAbs (such as E16, 5H10, 3A3, 7H2, and 5C5) are residues S^306^, K^307^, T^330^, and T^332^ within the DIII. These residues form a continuous patch on the solvent-exposed surface at the lateral tip of the WNV’s DIII (Volk et al., 2004). Any mutations in those epitopes result in the loss of neutralization function, strongly indicating the receptor binding sites of WNV (Beasley and Barrett, 2002; Volk et al., 2004). Therefore, sdAb_A1_ and sdAb_A6_, panned with a synthetic analog of DIII-1^L299–K307^, should have masked the residues S^306^ and K^307^ of rDIII to produce a blocking effect. Whether the other two residues T^330^ and T^332^ of rDIII are also masked by sdAb_A1_ and sdAb_A6_ is not claimed at the moment due to the possible difference in the secondary structure of rDIII produced in the *E. coli* expression system. The other two sdAbs, sdAb_A9_ and sdAb_A10_, could be recognizing the epitopes V^371^–R^388^ of DIII and/or a few more residues in their closest proximity. This prediction is based on the fact that rDIII ^L297–S403^ was used to coat the wells of the Ni plate, and the phages displaying sdAbs targeting three to four residues upstream or downstream of DIII-2^V371–R388^ could have been eluted. It is noteworthy that tripeptide R^388^-G^389^-E^390^ is in continuation with residues belonging to hBMEC receptor binding motif DIII-2^V371–R388^ (Supplementary Figure 1A). Arg-Gly-Asp/Arg-Gly-Glu-Ser (RGD/RGE) is a well-recognized motif for α_V_β_3_ integrin, acting as an important cell surface receptor prominently present in endothelial cells. Although contradictory reports exist on the importance of α_V_β_3_ integrins for the binding and entry of WNV in host cells models (*viz.* CS-1 melanoma cells and mouse embryonic fibroblast cell lines) and inhibiting the entry of virus by integrin specific antibodies effect (van der Most et al., 1999; Chu and Ng, 2004b; Chu et al., 2005; Schmidt et al., 2013; Edwards and Bix, 2019). Differences in their cell lines and antibodies could have resulted in varying outcomes (Schmidt et al., 2013). However, in this study, sdAb_A9_ and sdAb_A10_, which were found to be identical in their amino acid sequence (Supplementary Figure 2), were effectively blocking the rDIII-hBMEC interaction, and one of the possible reasons could be the masking of the R^388^-G^389^-E^390^ motif in rDIII. Whether the neutralizing effect of sdAb_A10_ (EC_50_ = 5709.86) on WNV-VLP (TCID_50_/mL = 8,100) is mainly due to interrupted entry of WNV-VLP into HEK293 cells (*in vitro*), the role of α_V_β_3_ integrins as possible mediators needs further study.

The sdAbs (sdAb_A1_, sdAb_A6_, and sdAb_A10_) were also tested for possible cross-reactivity with rDIII^ G301–K395^ of TBEV. Ideally, VNT should have been performed using either live TBEV or TBEV-VLPs. However, TBEV-VLPs were not commercially available, and it was not possible to handle the live virus in our BSL-2 laboratory. Therefore, ELISA was performed to determine the interaction between the sdAbs and rDIII of TBEV. The subtle cross-reactivity between sdAb_A1_/sdAb_A10_ and the rDIII of TBEV (Figure 6A) could be justified by the 34.2% identity in amino acid sequences of WNV-rDIII and TBEV-rDIIIs (Supplementary Figure 9) and the use of receptor binding motifs (synthetic analog of DIII-1^L299–K307^ and DIII-2^V371–R388^) to competitively elute the specific phages.

The VLPs used in the present study expressed the WNV’s E, C, and M protein on their surface, and the genome encoded just the luciferase, enabling the quantification of the infection on the basis of measured luminescence. VLPs are supramolecular multiprotein structures carrying all the necessary properties of viruses (Frazer, 2004; Pumpens, 2008; Zeltins, 2013). In particular, the VLPs used in the present study were designed to possess the identical antigens and symmetry on the surface epitopes of the native virus without the genetic code required for its replication (Mohsen et al., 2017). Therefore, WVN-VLPs was the appropriate alternative to live/virulent virus (with an obligation to work in the BSL3 laboratory) to evaluate the efficacy of developed sdAbs in our BSL2 laboratory (Nie et al., 2020).

It should be noted that, among the four sdAb clones (sdAb_A1_, sdAb_A6_, sdAb_A9_, and sdAb_A10_) that showed significant blocking of the interaction between rDIII and endothelial cells (Figure 3), sdAb_A1_ and sdAb_A6_ showed enormous precipitation during buffer exchange. Attempts were made to refold the aggregated sdAbs (data not shown) by solubilizing the precipitate in a higher concentration of 8 M urea followed by stepwise dialysis or addition of either oxidizing agents (DTT/β-mercaptoethanol), protein aggregation inhibitors (e.g., arginine) and protein stabilizers (e.g., PEG and glycerol) (Yamaguchi and Miyazaki, 2014), however, without success. As these efforts were out of the scope of this study, sdAb_A1_ and sdAb_A6_ were excluded from hemolytic and toxicity assays.

The sdAb_A10_ was tested for its hemocompatibility using sheep erythrocytes to evaluate its *in vivo* applicability as described in Rahman and Ochiai (2018). The incubation of sdAb_A10_ ranging from 5 μg (357 pmol) to 30 μg (2,14 nmol) with sheep erythrocytes resulted in just 0.1% of hemolysis (Figure 6B), which is very much lower than the maximum allowable hemolysis value of 5% for biomaterials (Autian, 1975). The positively charged sdAb_A10_ (estimated as +4.4) should have repelled with the positively charged erythrocyte membrane, thereby avoiding any electrostatic interaction and subsequent hemolysis (Slowing et al., 2009). Additionally, neutralizing sdAb_A10_ was also tested to determine its effect on the proliferation or viability of hBMEC due to possible cytotoxicity. The XTT assay employed in the present study evaluates cell viability based on cellular redox potential (Scudiero et al., 1988) and is an improvisation on the MTT assay generally used to evaluate the number of metabolically active cells (Levičar et al., 2003; Raghunandan et al., 2011). The viability of hBMEC in presence of sdAb_A10_ was 80% (Figure 6C), which is considered non-toxic as per the International Standards for Biological Evaluation of Medical Services [27].

## Conclusion

The sdAbs targeting the DIII domain of the WNV envelope glycoprotein were produced by phage display technology employing a single round of biopanning using hBMEC’s receptor binding motifs: DIII-1^L299–K307^ and DIII-2^V371–R388^. Among the four shortlisted sdAbs, sdAb_A10_ effectively blocked the interaction between rDIII and hBMECs *in vitro*. Further, sdAb_A10_ neutralized the WNV-VLP (TCID_50_/mL = 8100) exposed to HEK293 cell with an EC_50_ of 5709.86 (23 ng/ml) corresponding to 1.48 nM. Due to the absence of cytotoxicity and excellent hemocompatibility, sdAb_A10_ is a promising candidate for further preclinical trials and the development of specific anti-WNV therapy for humans.

## Data Availability Statement

The datasets presented in this study can be found in online repositories. The names of the repository/repositories and accession number(s) can be found in the article/Supplementary Material.

## Ethics Statement

The animal study was reviewed and approved by the Ethical Committee for handling animals of The University of Veterinary Medicine and Pharmacy in Košice, Slovakia, approved according to the regulations of Slovak government number 377/2012.

## Author Contributions

MB conceived the project and designed experiments. JH and AK conducted the experiments involving cloning, phage display, and sdAb generation. JČ did rearing, immunization, and blood collection from the llama. PP performed the production of recombinant DIII and toxicity assay. KB and JH performed the cell culture and virus neutralization test. EM and ZT performed the protein purification and sequencing. JH, AK, and MB prepared the manuscript. MB and AK received funding. All authors read and approved the final manuscript.

## Conflict of Interest

The authors declare that the research was conducted in the absence of any commercial or financial relationships that could be construed as a potential conflict of interest.

## Publisher’s Note

All claims expressed in this article are solely those of the authors and do not necessarily represent those of their affiliated organizations, or those of the publisher, the editors and the reviewers. Any product that may be evaluated in this article, or claim that may be made by its manufacturer, is not guaranteed or endorsed by the publisher.
