# Supplementary material for: Engineering the Single Domain Antibodies Targeting Receptor Binding Motifs Within the Domain III of West Nile Virus Envelope Glycoprotein
Source: Front Microbiol. 2022 Apr 1;13:801466. doi: 10.3389/fmicb.2022.801466 (PMC9012491; doi:10.3389/fmicb.2022.801466)
Supplement: Supplementary file 1 [file Data_Sheet_1.DOCX]

## **Engineering the single domain antibodies targeting receptor binding motifs within the domain III of West Nile virus envelope glycoprotein**

**Jana Hruškovicová^1^, Katarína Bhide^1^, Patrícia Petroušková^1^, Zuzana Tkáčová^1^, Evelína Mochnáčová^1^, Ján Čurlík^2^, Mangesh Bhide^1,3^ and Amod Kulkarni^1,3*^**

^1^Laboratory of Biomedical Microbiology and Immunology, The University of Veterinary Medicine and Pharmacy, Košice, Slovakia,

^2^Department of breeding and diseases of game, fish and bees, ecology and cynology, The University of Veterinary Medicine and Pharmacy, Košice, Slovakia

^3^Institute of Neuroimmunology, Slovak Academy of Sciences, Bratislava, Slovakia.

# Supplementary figures


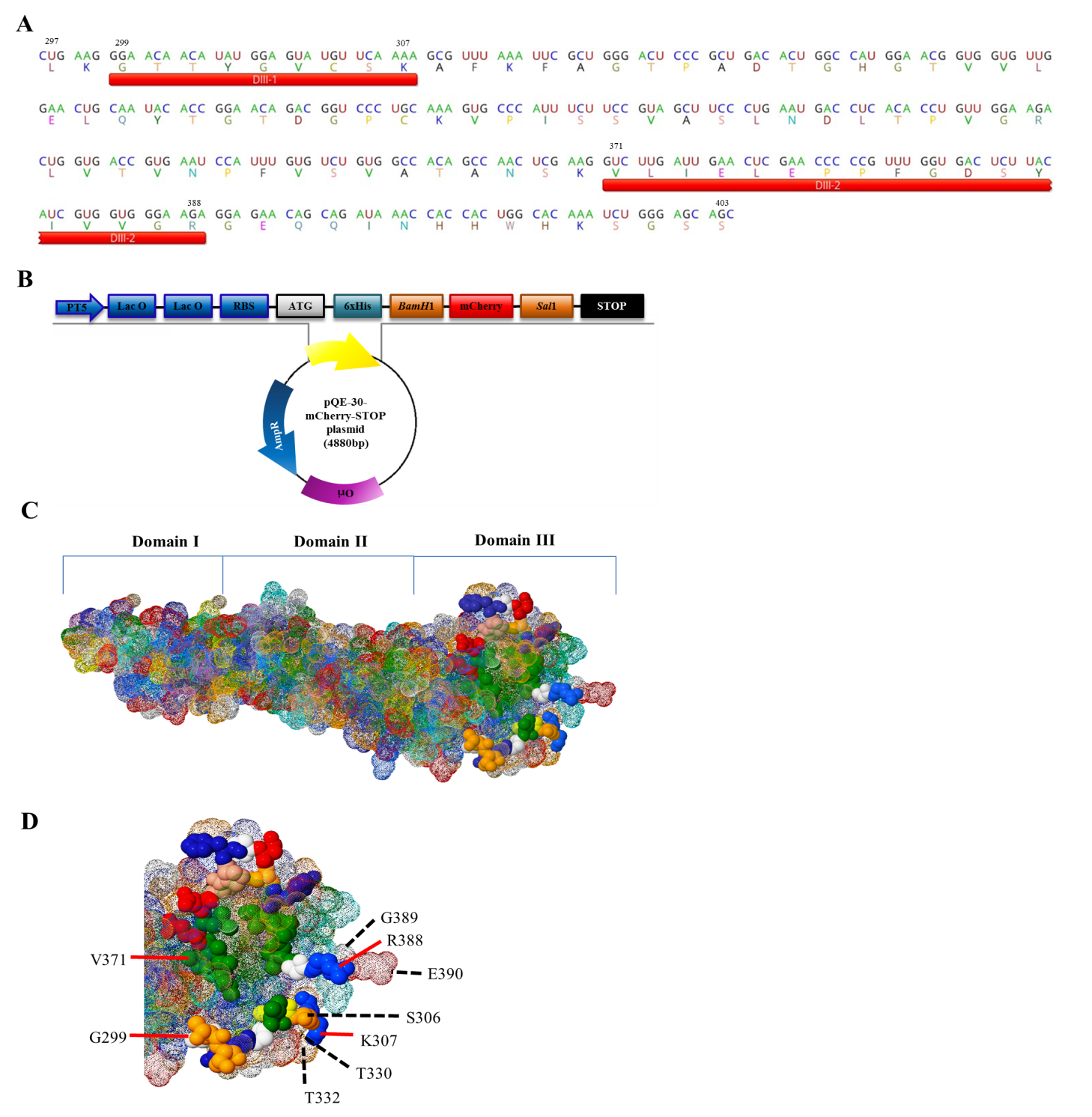


**Figure S1: Sequence of DIII used for production of recombinant form and its structure. (A)** Sequence of DIII of WNV (Goshawk, Genbank: DQ116961.1). Red annotations show receptor binding motifs DIII-1^G299-K307^ and DIII-2^V371--R388^. **(B)** Vector map of pQE-30-mCherry-STOP used to produce rDIII of WNV. Sequence of mCherry (stuffer) flanking the *BamH*I and *Sal*I site was digested to replace it with DIII ^L297–S403^ sequence amplified by PCR. T5-promotor; Lac O-lac operon; RBS-ribosome binding site; ATG-start codon; His-his tag sequence; STOP-stop codon; AmpR-gene of ampicillin resistance; Ori-origin of replicaton. **(C)** Crystal structure of E glycoprotein of WNV (PDB accession number 2HG0) showing all three domains. **(D)** Enlarged structure of DIII with amino acid residues highlighted within the receptor binding motifs. Receptor binding motifs DIII-1^G299-K307^ and DIII-2^V371-R388^ are highlighted. Residues forming the start and end of receptor binding motifs are indicated with red lines. Other residues involved in the cell surface receptor interaction are denoted by dotted lines.


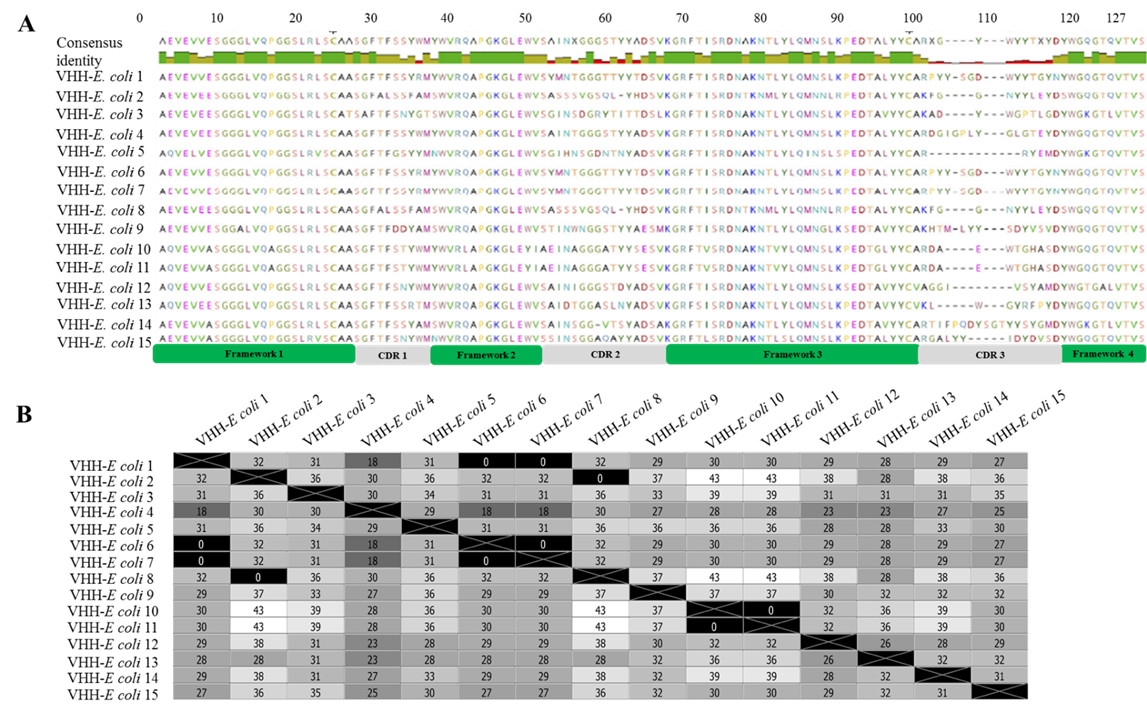


**Figure S2: Amino acid sequences of transformants belonging to VHH-*E. coli* library assessed for their diversity. (A)** Amino acid sequences of 15 randomly selected transformants belonging to VHH-*E. coli* library were aligned with reference VHH sequences and the frameworks regions 1-4 and CDR (complementarity-determining regions) 1-3 were marked. The consensus sequence shows high similarity in frameworks regions (1-4) and variation in CDR (1-3). **(B)** Distance matrix plot on the aligned sequences in A denotes variation in amino acid residues (mentioned in boxes) indicated by various shades of gray within the compared sequences. White color represents the highest difference between the two compared clones, while black color represents no difference.


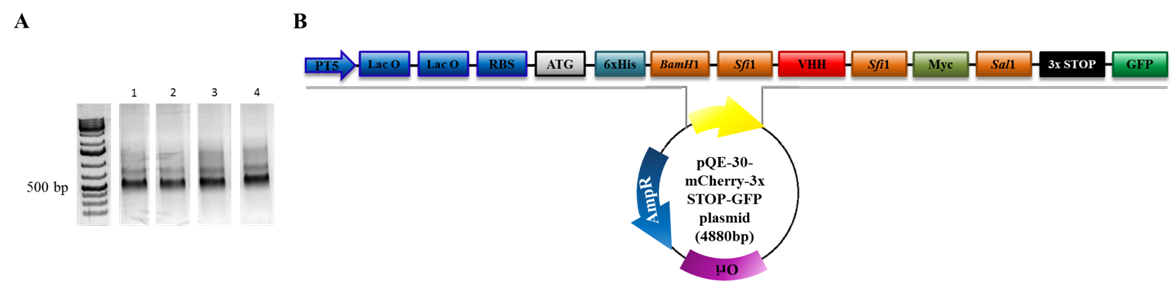


**Figure S3: Amplification of VHH from eluted phages from panning and vector map of pQE-30-UA-mCherry-3x Stop-GFP. (A)** VHH (~500 bp) amplified from the DNA of phages eluted after biopanning with DIII-1^G299-K307^ (lane 1 and 2) and DIII-2^V371-R388^ (lane 3 and 4) using VHH-F and VHH-R primers. **(B)** Vector map of pQE-30-mCherry-3xSTOP-GFP used to ligate the *Sfi*I digested VHH excised from panel A. T5-promotor; Lac O-lac operon; RBS-ribosome binding site; ATG-start codon; His-his tag sequence; *BamH*I-restriction site; *Sfi*I-restriction site; VHH-gene of sdAb; Myc-myc tag sequence; STOP-stop codon; GFP-Green fluorescent protein; AmpR-gene of ampicillin resistance; Ori-origin of replication.


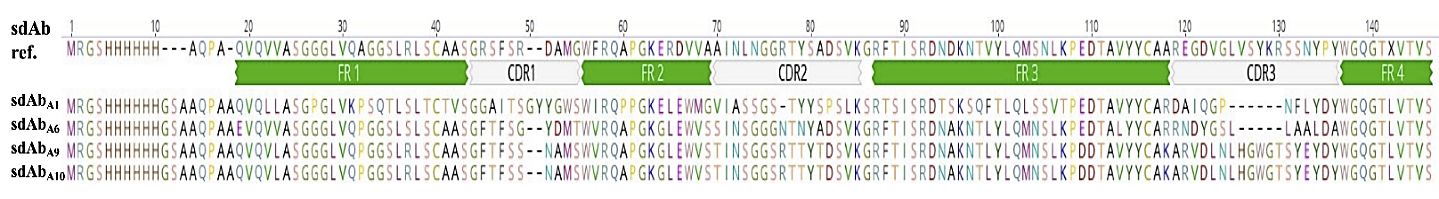


**Figure S4: The amino acid sequence of overexpressed sdAbs.** The sequences of sdAbs were aligned to sdAb reference sequence. Regions of FR (frameworks) and CDRs (complementarity-determining regions) are marked.

**
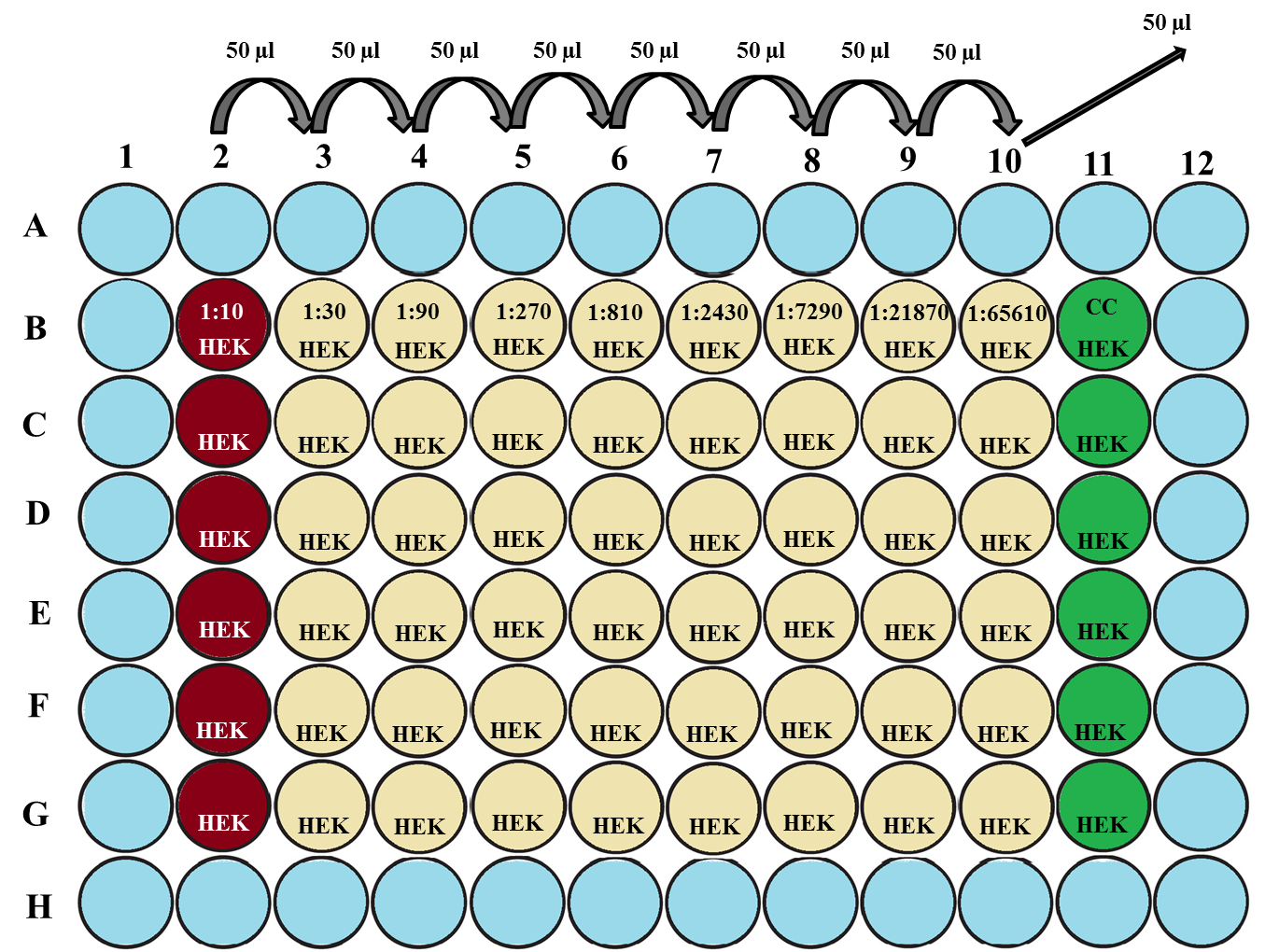
**

**Figure S5: Titration of WNV-VLP**. The blue wells on the periphery of 96 well plate contain sterilized water (no cells). Red wells were added with the primary dilution of WNV-VLP (1:10): 15 μl WNV-VLP in 135 μl DMEM. Yellow wells represent 3-fold serial dilutions that were made as follows: 100 ul DMEM was added to each yellow well (column 3-11). After adding the WNV-VLP of the red wells (B2-G2), the contents of the wells were mixed, and then 50 μl was transferred to column 3 (B3-G3). The content of the wells in column 3 was mixed and 50 μl from column 3 was transferred to column 4 (B4-G4). The procedure was repeated until column 10 (B10-G10), from where 50 μl were discarded. Green wells represent a cell control (CC) that contains only 150 μl DMEM without WNV-VLP.

**
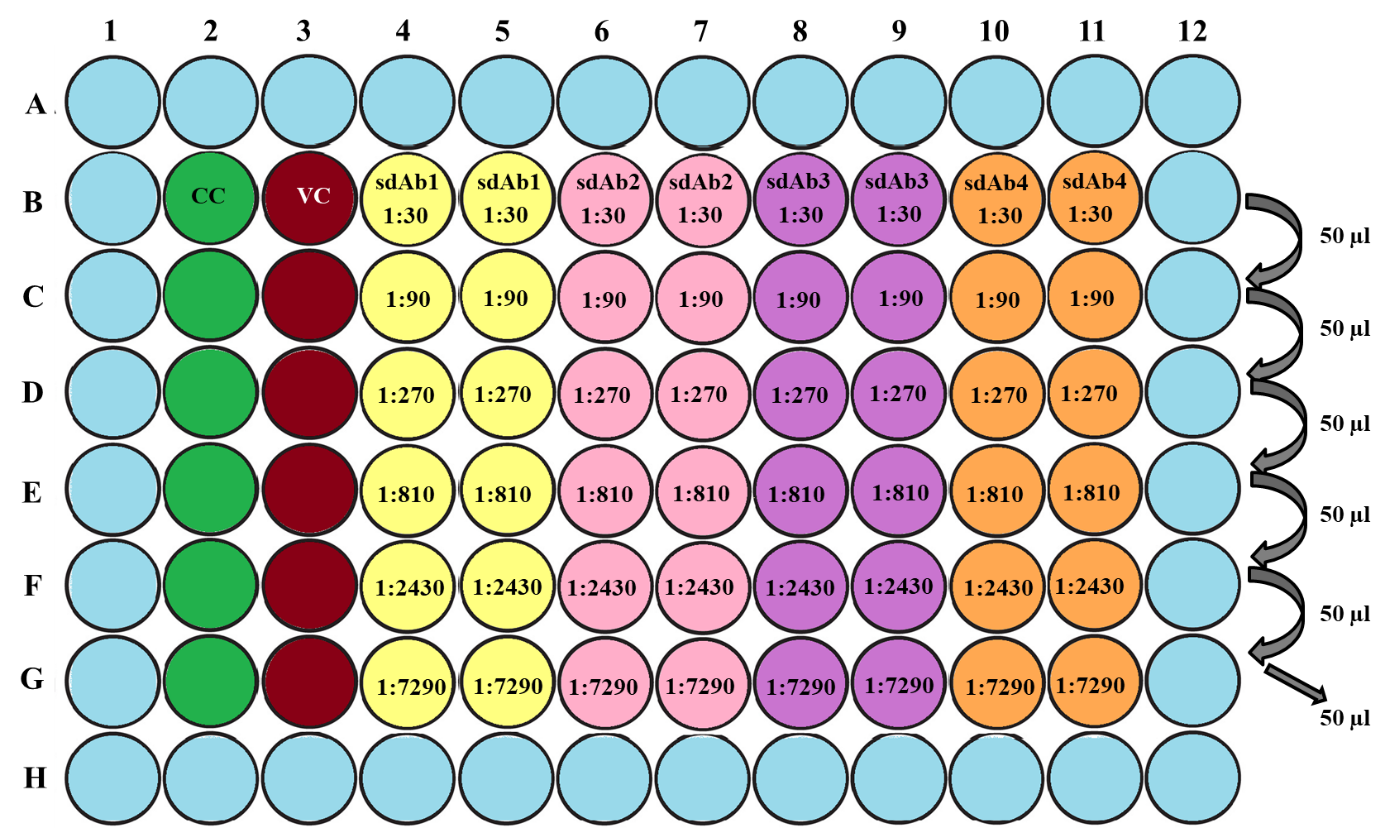
**

**Figure S6**: **Dilution plate**. The blue wells on the periphery contain only water. Green wells represent cell control (CC) having HEK293/17 cells with only 150 μl DMEM and no WNV-VLP or sdAb. Red wells serve as virus control (VC) where in HEK293/17 cells are exposed to WNV-VLP (400-500 TCID_50_/ml). Wells in column 4 to 11 were used to add 3-fold serial dilutions of sdAbs in duplicate (i.e VNT on 4 sdAbs can be tested in one plate). Note that, 3 fold diluted hyper immune serum of horse surviving natural WNV infection (positive control) or 3 fold diluted non-related sdAb (negative control: VHH_F3_ raised against *Neisseria meningitidis*) were substituted in place of sdAb2 and sdAb4 respectively to perform the entire assay in multiple plates.

**Figure S7: Ability of sdAb_A10_ to neutralize WNV-VLP.** Ability of 3 fold diluted sdAb_A10_ to neutralize WNV-VLP until 9 dilutions is depicted where in the initial dilution used in the assay was 30. It is noteworthy that beyond 5 dilutions (dilution factor = 7290) of sdAb_A10,_ there was drastic decrease in neutralization of WNV-VLP.

**
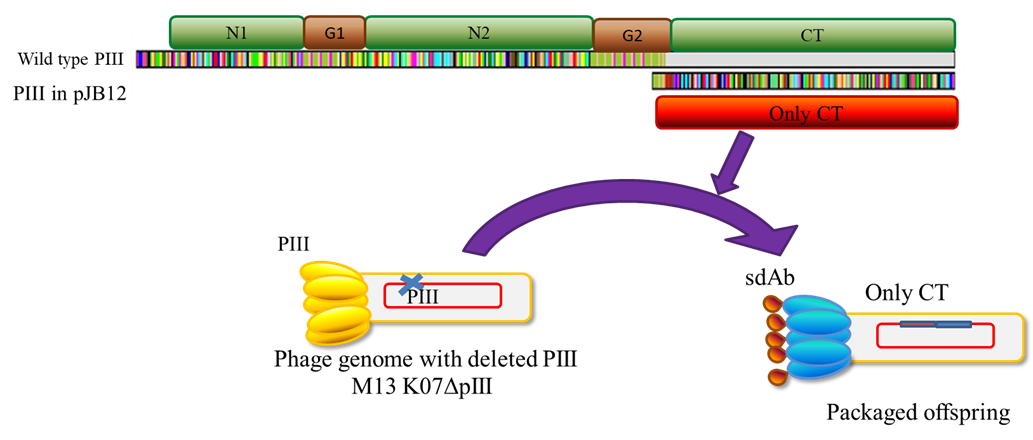
**

**Figure S8: Structure of non-infective Hyperphage M13 K07ΔpIII.** The Hyperphage M13 K07ΔpIII used in this study does not contain the gene encoding PIII in its genome (only the promoter and signal peptide are retained) but just the phenotypically expressing PIII (yellow ovals). Wild type PIII gene consists of N1, N2, and CT domains intermediated by the G1 and G2 glycine-rich linkers. N1 and N2 domains of PIII are required to bind TolA surface protein and the F pilus of *E. coli* respectively to mediate the infection. Whereas the CT domain is essential for correct assembly of phages and their release from *E. coli* during phage replication. G1, G2 linkers provide flexibility to nearby domains during infection. In the present study, VHH-*E. coli* library was transformed with phagemid pJB12 carrying VHH fused to the N terminus of supershort version of PIII (CT domain only). Thus the escaped phages will display sdAbs on truncated PIII i.e. on CT domain (brown ovals) and do not possess the N1 and N2 domains which will demerit their ability to interact with *E. coli* and causes infection.

**
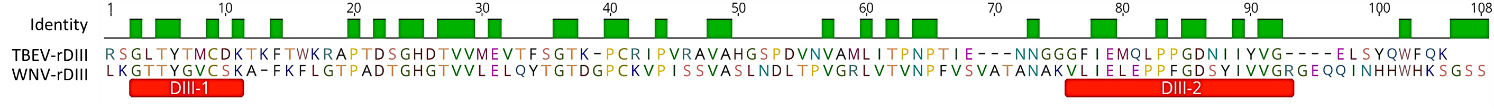
Figure S9: Alignment of amino acid sequences.** Alignment of amino acid sequences of TBEV-rDIII (rDIII ^G301-K395^, Hypr strain, accession no: **2022145A**) and WNV-rDIII (rDIII ^L297-S403^ Goshawk strain accession no. **YP_001527880**) used to immunize the llama. Red annotations denote receptor binding motifs of WNV-rDIII used in competitive elution of specific phages during phage display.

Supplementary Table 1 Synthetic analogs of receptor binding motifs of DIII

| **Peptide name** | **Sequence** | **Mol. mass of the biotinylated synthetic peptide [Da]** |
| --- | --- | --- |
| DIII-1^G299-K307^ | GTTYGVCSK-biotin | 1142.26 |
| DIII-2^V371-R388^ | VLIELEPPFGDSYIVVGRK-biotin | 2356.80 |

# Supplementary Methods

#

**Supplementary Method 1: Amplification of VHH from peripheral blood mononuclear cells, and generation of VHH-*E. coli* library.**

100 ml of heparinized blood collected from immunized llama was subjected to isolation of peripheral blood mononuclear cells (PBMC) by density gradient centrifugation using Histopaque medium (Sigma-Aldrich) and the protocol described by the manufacturer. RNA was extracted from isolated PBMCs using the RNeasy mini kit (Qiagen) which includes a step to eliminate genomic DNA. RevertAid (Thermo Scientific, Slovakia) and gene specific primer: VHH-Not-R (**Table 1**) was used to reverse transcribe ~5μg of RNA into cDNA.

VHH region flanking the frameworks 1 to 4 was amplified by PCR using degenerate primers VHH-F and VHH-R (**Table 1**) and 500 ng of template cDNA. PCR conditions were set as 95 °C-2 min and 25 cycles of 95 °C-20 s, 56 °C-30 s, 68 °C-1 min and 68 °C-10 min. It is noteworthy that the PCR cycles were restricted to 25 to achieve diversified sdAbs as opposed to amplification of single species of sdAbs. The resulting amplicon (~500 bp) was gel purified (Macherey-Nagel, Germany), digested with *Sfi*I (Thermo Fisher Scientific) at 50 °C for 1 h and ligated into *Sfi*I digested phagemid pJB12. The ligation mix was electroporated into *E. coli* XL-1 blue (New England Biolabs, Germany) using the preset method for *E. coli* - voltage: 1.8 KV, capacitance: 25 μf and resistance 200 Ω in Gene Pulser X cell (Biorad, UK). Transformed *E. coli* from the 19 electroporations were preliminarily cultured in SOC medium for 1h at 37 °C and plated on six LB agar (Sigma-Aldrich) plates (diameter 18.5 cm) containing tetracycline (50 μg/ml; Duchefa Biochemie BV), and chloramphenicol (50 μg/ml; Duchefa Biochemie BV). Plates were incubated at 37 °C for 16 h and the bacterial colonies were scraped and stored as VHH-*E. coli* library in the aliquots of 10 ml LB medium containing glycerol (50%; MikroChem) at -80 °C until further use. Diversity of VHH sequence within the cloned *E. coli*, was assessed by sequencing fifteen randomly selected transformants (**Figure S2**) using primers UA-insertom-His-sense and UA-insertom-GFP-antisense (**Table 1**).

**Supplementary Method 2: Titration of WNV-VLP**

Titration of WNV-like particles (WNV-VLP) was performed according to the recently published protocol [2] with minor modification. In brief, HEK293/17 cells were seeded (2x10^4^ per well, passage 11) on 60 wells of 96 well plate (column 2 to 11 in **Figure S5**) containing 200 µl of DMEM-GlutaMAX complete medium (Dulbecco's Modified Eagle Medium-GlutaMAX basal medium supplemented with 10% fetal bovine serum, 2 mM of L-glutamin, Gibco) and incubated overnight at 37 °C in CO_2_ (5%). The outer most wells were filled with sterile water (no cells). The titration plate was prepared as follows: Wells in column 2 served as primary dilution of WNV-VLP (1:10) where 135 µl of DMEM-GlutaMAX complete medium and 15 µl of WNV-VLP was added. Next columns (3-10) were added with 3-fold serial dilutions: 100 μl of DMEM-GlutaMAX complete medium was added to each well. After adding WNV-VLP in column 2, the content of wells was mixed and 50 μl was transferred to column 3. The content of wells in column 3 was mixed and 50 μl was transferred to column 4. The procedure was repeated until column 10, and 50 μl from the wells of column 10 was discarded. Wells in column 11 (CC) served as negative control wherein addition of WNV-VLP is omitted.

The medium from overnight grown culture was discarded carefully and the content from each wells of the titration plate (**Figure S5**) was transferred to culture plate containing HEK293/17 cells. The plate was incubated for 48 h at 37 °C and 5% CO_2_ followed by emptying the culture medium and lysing the HEK293/17 cells with 20 µl of Luciferase Cell Culture Lysis 1X Reagent (Promega, USA). Next, the lysates were transferred into opaque 96 well plate (Promega), and 100 µl of the luminescent substrate was added before detecting the luminescence on Cytation7 imaging system (BioTek, U.S.A.). Parameters on Cytation7 were set as integration time-10 sec, read height 5.4 mm and the gain 240. To determine the VLP titer, values obtained from Cytation7 were copied in the template in excel (**Supplementary Data Sheet 2**). This template is adopted from recently published paper [2].

**Supplementary Method 3: Vitus neutralization test**

HEK293/17 cells were seeded (13 passage, 2x10^4^ per well) on 60 wells of 96 well plate in column 2 to 11, containing 200 µl of DMEM-GlutaMAX complete medium whereas peripheral wells were filled with sterile water (no cells). Cells were cultured overnight as described above.

Next day, the dilution plate (**Figure S6**) was prepared to pre-incubated sdAbs with WNV-VLP. In 96 well plate, 6 wells (B2-G2) served as cell control (CC) in which 150 μl of DMEM-GlutaMAX was added (sdAbs and WNV-VLP were excluded). The other 6 wells (B3-G3) served as a virus control, where 100 μl of DMEM-GlutaMAX and 50 μl of WNV-VLP (400-500 TCID_50_/ml) were added. Wells B4-G11 (in duplicates) were used for diluting sdAbs. (Note that inactivated hyperimmune serum can be substituted with sdAbs in duplicate wells to generate positive control for e.g. wells B6-G7). 142.5 μl DMEM-GlutaMAX complete medium (without antibiotics) was added to wells in row B4 to B11, while 100 μl DMEM-GlutaMAX complete medium (without antibiotics) was added to remaining wells (C4-G11). sdAbs were 3-fold serially diluted in the following manner: 1 μg of sdAb (maximum volume - 3.2 μl) resupended in 1% inactivated normal serum (final volume = 7.5 μl) was added in top wells (B4 to B11) and mix the contents. 50 μl from wells in row B were transferred to row C and the procedure was continued until row G. The extra 50 μl in wells of row G was discarde. After creating the dilution series, 50 μl of WNV-VLP (400-500 TCID_50_/ml) were added to all the wells except in cell control and the pre-incubation was continued for 90 min at 37 °C in 5% CO_2_ incubator.

The medium from overnight grown HEK293/17 cell culture plate was discarded just before the initiation of virus neutralization test (VNT). Subsequently, the contents from the dilution plate were transferred to the corresponding wells in the HEK293/17 plate and incubation was carried out for 48 h at 37 °C in 5% CO_2_ incubator. After incubation, the medium from all the 60 wells were discarded and the lysis of HEK293/17 was performed by adding 20 µl of Luciferase Cell Culture Lysis 1X Reagent (Promega). The resulting lysate was transferred to opaque 96 well plates (Promega), and 100 µl of the luminescent substrate was added. Thereafter the luminescence was detected on the Cytation7 imaging system (integration time-10 sec, read height 5.4 mm and the gain 240, BioTek). The readings for luminescence were exported in the excel template (**Supplementary Data Sheet 3 and 4**) to calculate EC_50._ This template is adopted from recently published paper [2].

**References**

1. Schaefer J.V., Honegger A. and Pluckthun A. Construction of scFv Fragments from Hybridoma or Spleen Cells by PCR Assembly. Antibody Engineering, Vol 1, Second Edition:21-44, 2010.

2. Nie J., Li Q., Wu J., Zhao C., Hao H., Liu H., Zhang L., Nie L., Qin H., Wang M., Lu Q., Li X., Sun Q., Liu J., Fan C., Huang W., Xu M. and Wang Y. Quantification of SARS-CoV-2 neutralizing antibody by a pseudotyped virus-based assay. Nat Protoc 15(11):3699-3715, 2020.
