# Supplementary material for: Engineering the Single Domain Antibodies Targeting Receptor Binding Motifs Within the Domain III of West Nile Virus Envelope Glycoprotein
Source: Front Microbiol. 2022 Apr 1;13:801466. doi: 10.3389/fmicb.2022.801466 (PMC9012491; doi:10.3389/fmicb.2022.801466)
Supplement: Supplementary file 5 [file Presentation_1.PPTX]

## Slide 1
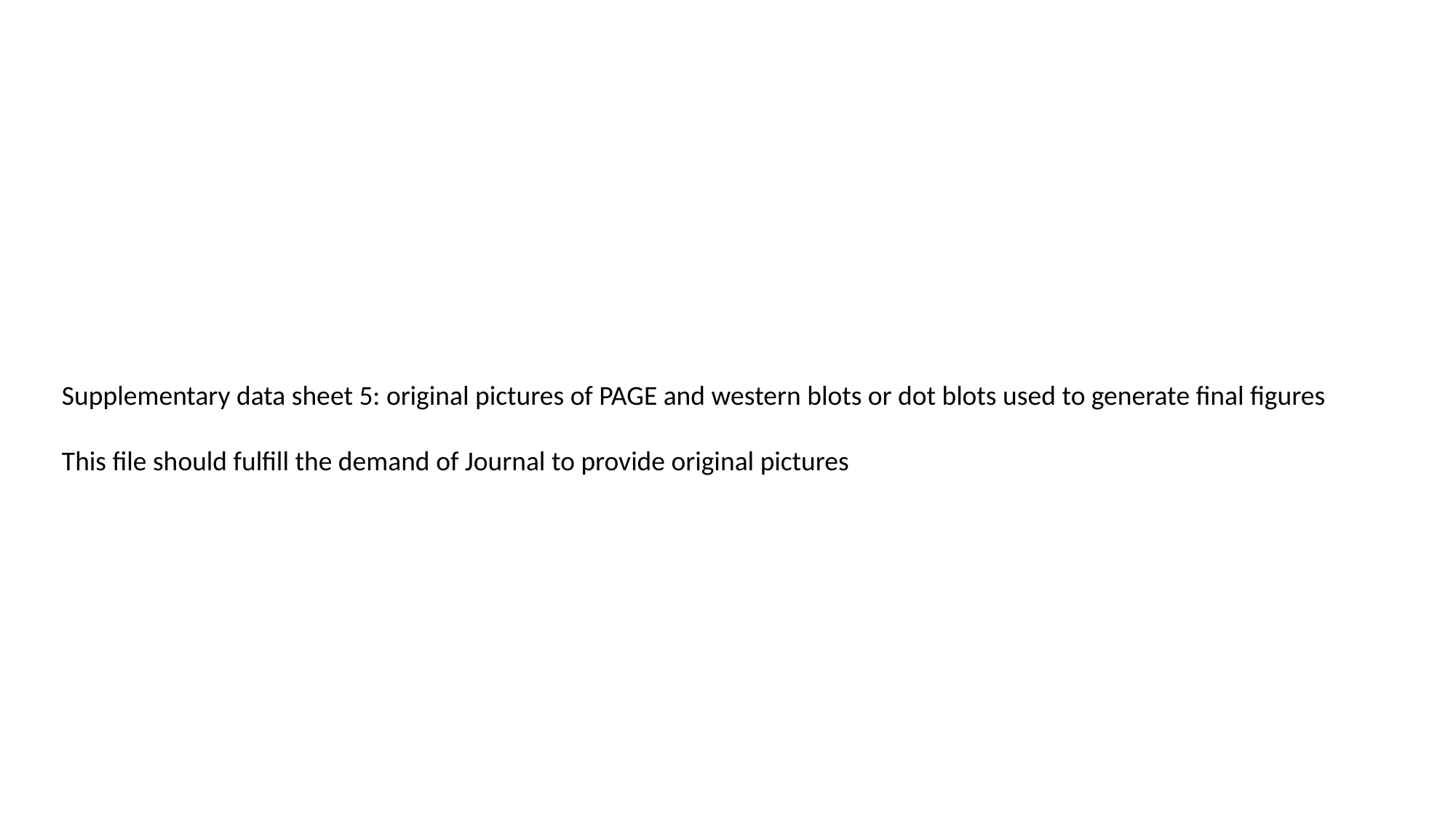

Supplementary data sheet 5: original pictures of PAGE and western blots or dot blots used to generate final figures
This file should fulfill the demand of Journal to provide original pictures

## Slide 2
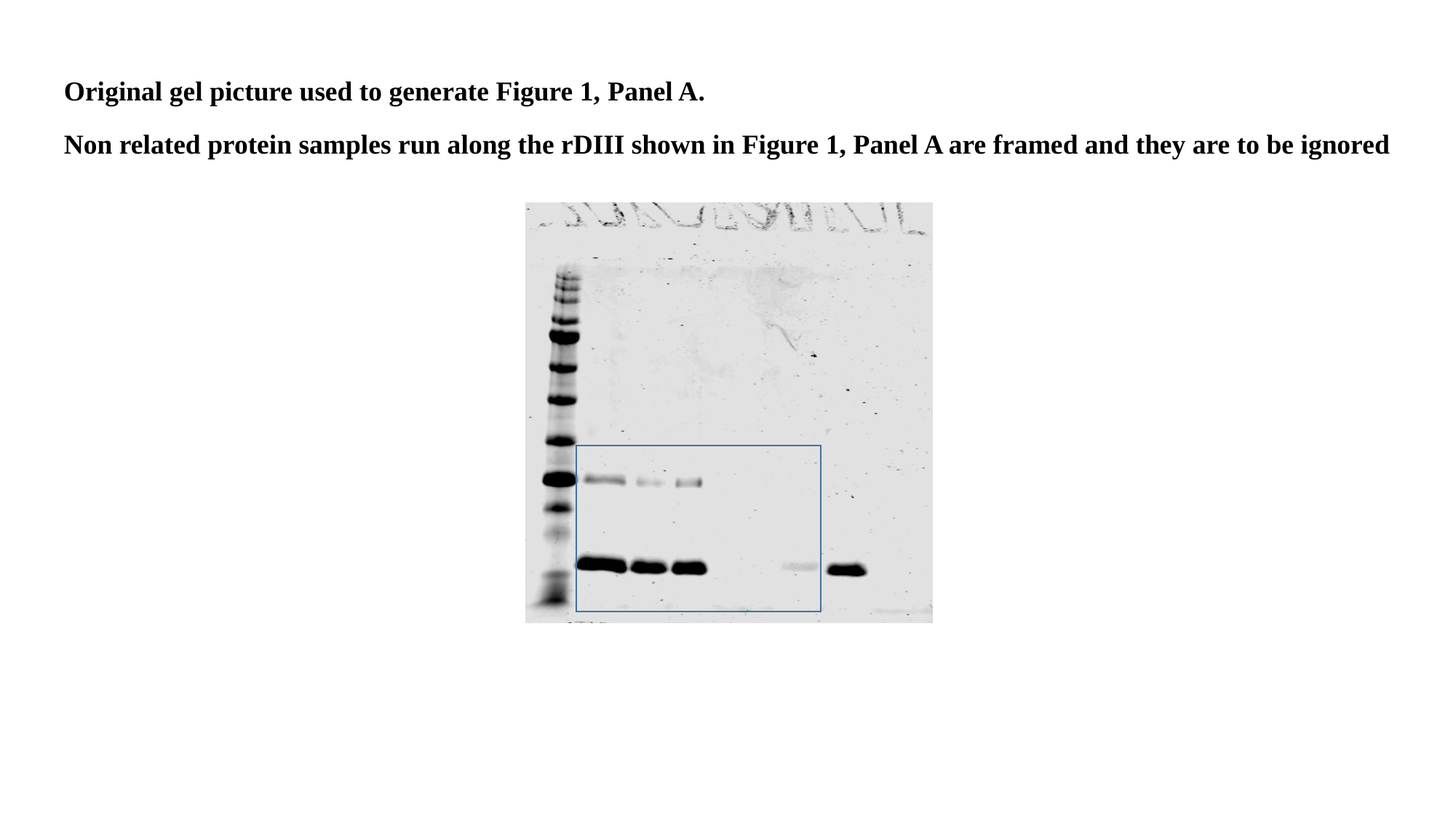

Original gel picture used to generate Figure 1, Panel A.
Non related protein samples run along the rDIII shown in Figure 1, Panel A are framed and they are to be ignored

## Slide 3
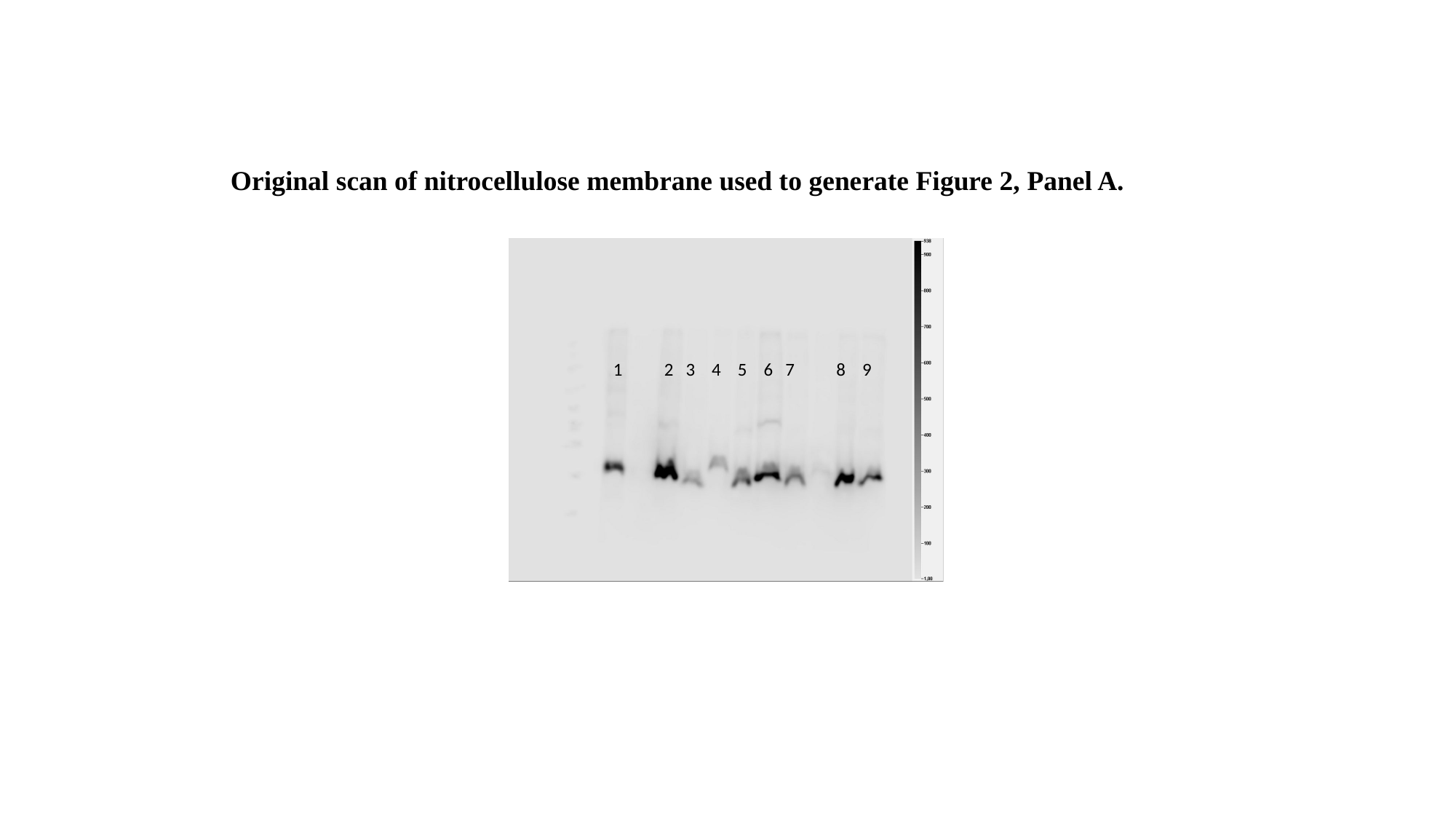

Original scan of nitrocellulose membrane used to generate Figure 2, Panel A.
1 2 3 4 5 6 7 8 9

## Slide 4
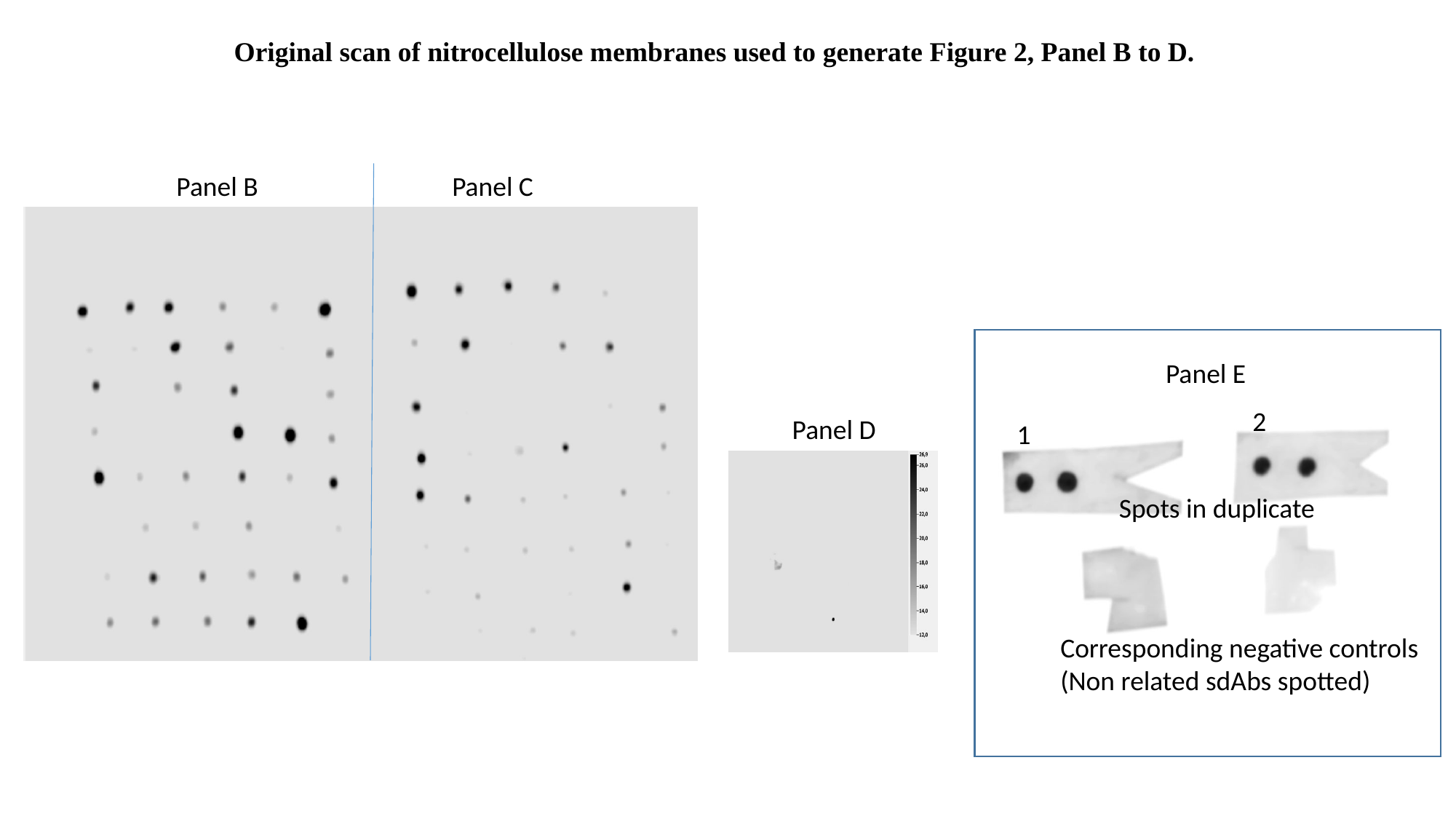

Original scan of nitrocellulose membranes used to generate Figure 2, Panel B to D.
Panel B
Panel C
Panel E
2
Panel D
1
Spots in duplicate
Corresponding negative controls
(Non related sdAbs spotted)

## Slide 5
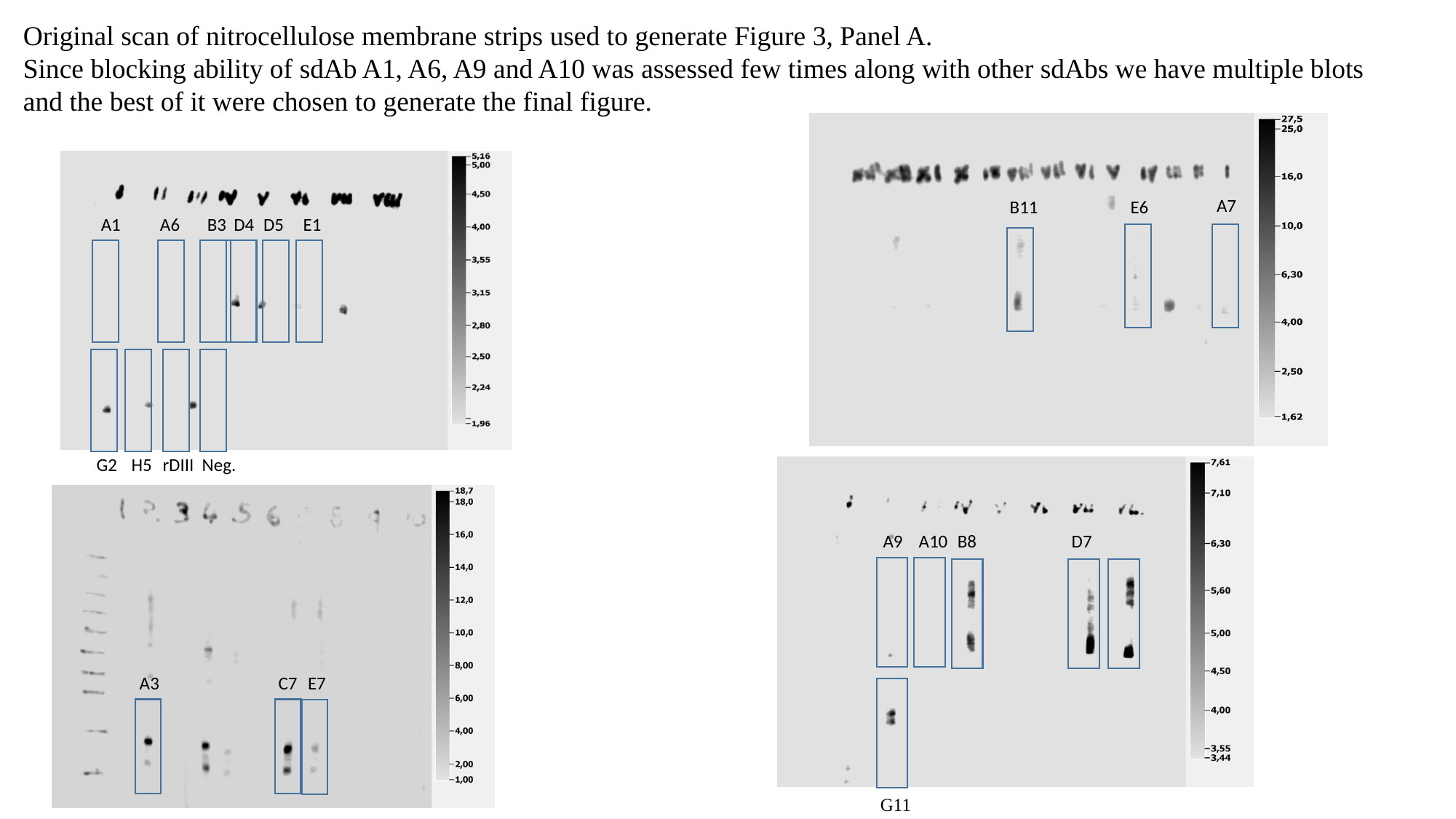

Original scan of nitrocellulose membrane strips used to generate Figure 3, Panel A.
Since blocking ability of sdAb A1, A6, A9 and A10 was assessed few times along with other sdAbs we have multiple blots and the best of it were chosen to generate the final figure.
A7
E6
B11
A1
A6
B3
D4
D5
E1
Neg.
rDIII
H5
G2
A9
A10
B8
D7
A3
C7
E7
G11

## Slide 6
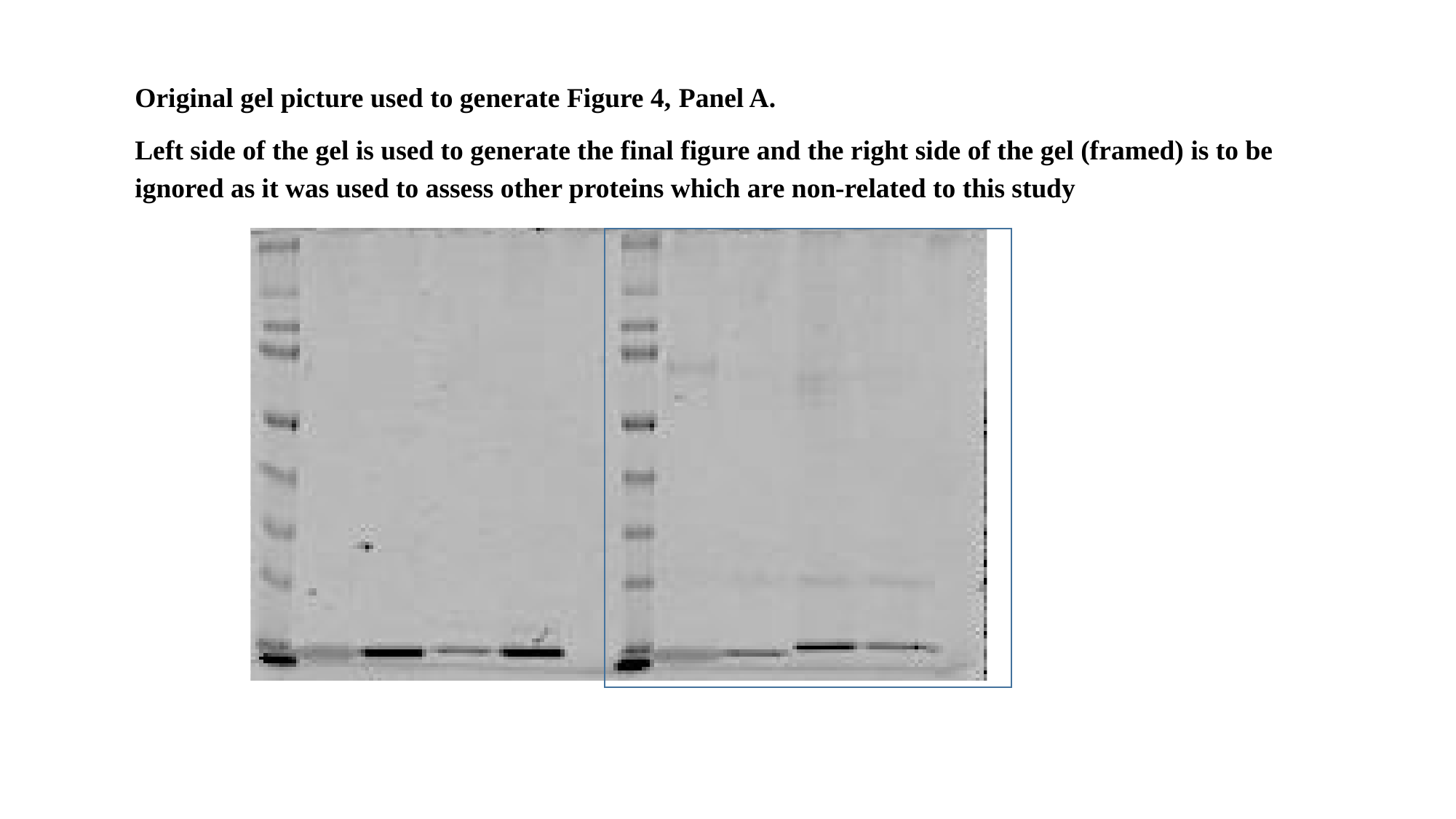

Original gel picture used to generate Figure 4, Panel A.
Left side of the gel is used to generate the final figure and the right side of the gel (framed) is to be ignored as it was used to assess other proteins which are non-related to this study
